# Quantifying the Kinetics of Signaling and Arrestin Recruitment by Nervous System G-Protein Coupled Receptors

**DOI:** 10.3389/fncel.2021.814547

**Published:** 2022-01-17

**Authors:** Sam R. J. Hoare, Paul H. Tewson, Shivani Sachdev, Mark Connor, Thomas E. Hughes, Anne Marie Quinn

**Affiliations:** ^1^Pharmechanics LLC, Owego, NY, United States; ^2^Montana Molecular, Bozeman, MT, United States; ^3^Department of Biomedical Sciences, Macquarie University, Sydney, NSW, Australia

**Keywords:** arrestin, biosensor, cannabinoid, dynamics, G protein coupled receptor (GPCR), kinetics, opioid, partial agonist

## Abstract

Neurons integrate inputs over different time and space scales. Fast excitatory synapses at boutons (ms and μm), and slow modulation over entire dendritic arbors (seconds and mm) are all ultimately combined to produce behavior. Understanding the timing of signaling events mediated by G-protein-coupled receptors is necessary to elucidate the mechanism of action of therapeutics targeting the nervous system. Measuring signaling kinetics in live cells has been transformed by the adoption of fluorescent biosensors and dyes that convert biological signals into optical signals that are conveniently recorded by microscopic imaging or by fluorescence plate readers. Quantifying the timing of signaling has now become routine with the application of equations in familiar curve fitting software to estimate the rates of signaling from the waveform. Here we describe examples of the application of these methods, including (1) Kinetic analysis of opioid signaling dynamics and partial agonism measured using cAMP and arrestin biosensors; (2) Quantifying the signaling activity of illicit synthetic cannabinoid receptor agonists measured using a fluorescent membrane potential dye; (3) Demonstration of multiplicity of arrestin functions from analysis of biosensor waveforms and quantification of the rates of these processes. These examples show how temporal analysis provides additional dimensions to enhance the understanding of GPCR signaling and therapeutic mechanisms in the nervous system.

## Introduction

The timing of molecular events is central to the orchestration of cellular activity that underlies the functions of the nervous system. Quantifying activity over time, from the action potential to synaptic plasticity to neural oscillations, has been essential to understand cellular physiology in neuroscience research. Recent advances have greatly expanded the temporal understanding of G-protein-coupled receptor activity (GPCR) in the nervous system. A new class of genetically encoded, fluorescent biosensors make it possible to image the release, spread, and clearance of important neurotransmitters and modulators in the extracellular space (Marvin et al., 2013; Patriarchi et al., 2018; Unger et al., 2020). In turn the stimulation of the GPCRs and subsequent intracellular signaling pathways can now be studied in real time, capturing the kinetics and spatial distribution of the signaling that the neurotransmitters provoke (Ferrandon et al., 2009; Zhao et al., 2011; Lohse et al., 2012; Vilardaga et al., 2014; Irannejad et al., 2015; Ohno et al., 2017; Greenwald et al., 2018; Halls and Canals, 2018; Jullie et al., 2020; Olsen et al., 2020; Kuroda et al., 2021; Wright and Bouvier, 2021; Zhang et al., 2021b). We are moving beyond the question of whether signaling has occurred to a new era in which we can watch in real time the exact nature of where and when these important events occur.

Until recently, the timing of GPCR signaling was not routinely measured [with the notable exception of GPCR regulation of ion channel activity (Suh et al., 2004; Johnson et al., 2005)]. For example, for the cAMP signaling pathway, it was typical to measure this response at only a single time point, using end-point assays in which the cells are lysed and the signal analyte measured chemically or with antibody-based detection methods. Such single time point (“Endpoint”) assays have been used overwhelmingly in GPCR research. End-point assays measure the summed outputs of signaling from activated, desensitized and internalized receptors while providing little insight into the real-time dynamics of receptor activation, and this may have a profound influence on the detection and interpretation of receptor signaling by different drugs (Suh et al., 2004; Charlton and Vauquelin, 2010; Klein Herenbrink et al., 2016; Bdioui et al., 2018; Hoare et al., 2018, 2020b; Zhao and Furness, 2019; Zhu et al., 2019; Finlay et al., 2020).

**Figure 1:**
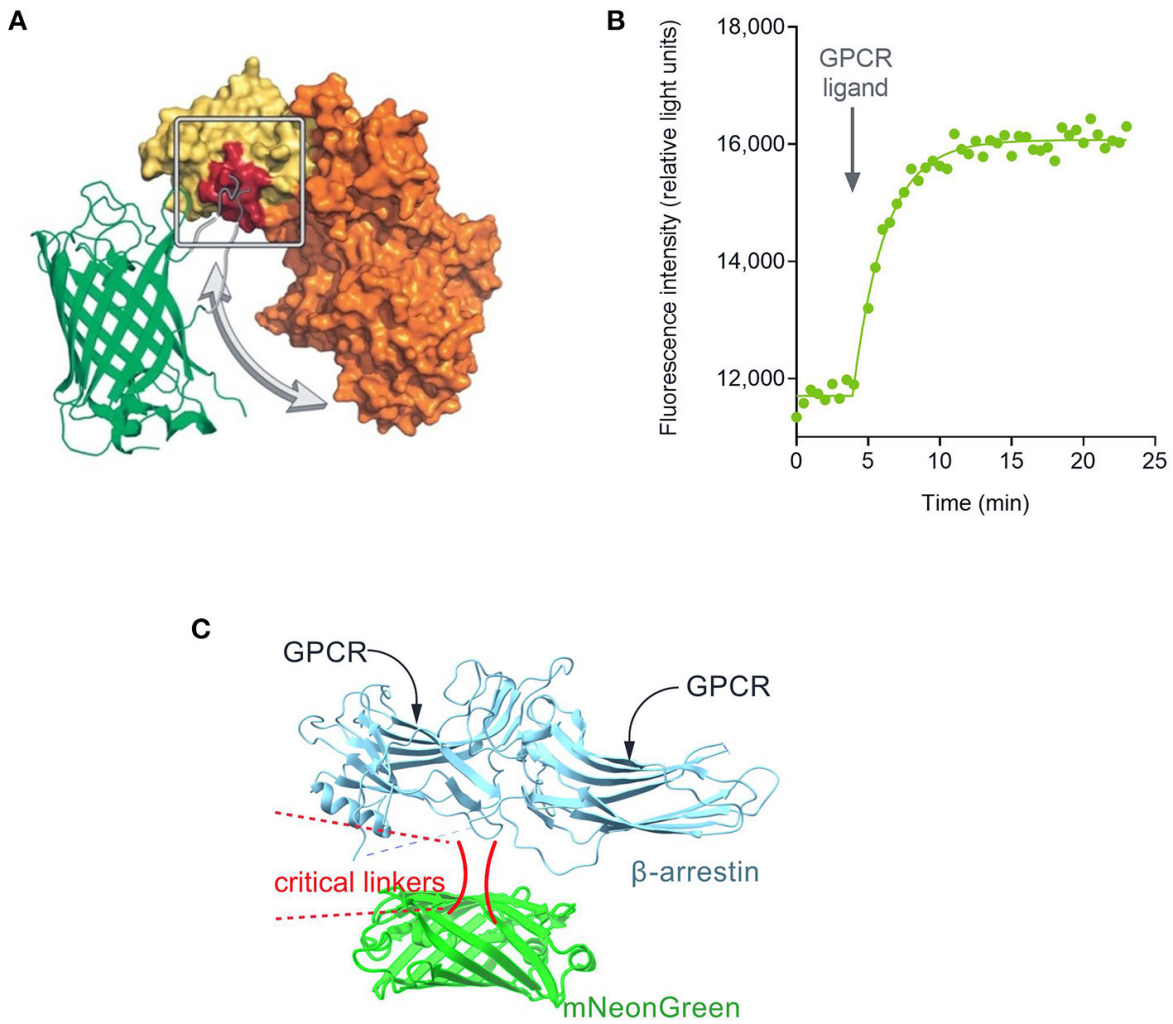
Fluorescent biosensor architecture and function, illustrated using the cADDis cAMP sensor (Tewson et al., 2016). **(A)** Direct fluorescent biosensors comprise a fluorescent protein (in this case mNeon Green) coupled to a protein that binds the analyte to be detected (in this case EPAC2 binding cAMP). Binding of cAMP to the biosensor causes a change in conformation that results in a change in the optical properties of the fluorescent protein. **(B)** This change, in this case a change of fluorescence emission intensity, can be recorded in a fluorescence plate reader. **(C)** Architecture of fluorescent arrestin sensor employed in this study. Arrestin-3 was engineered to incorporate mNeonGreen, as described (Hoare et al., 2020a). Interaction with the receptor causes a change in fluorescence intensity that can be detected in a plate reader.

Quantifying the whole time course of signaling has now been enabled with the development of sensors that convert the biological signal into a light signal which can be recorded repeatedly or continuously in live cells. The first such reagents developed were the calcium indicators, chemical dyes that change in fluorescence on binding calcium (Minta et al., 1989). This paradigm has also been applied to detect other signals, such as changes of voltage (Waggoner, 1979). The study of signaling kinetics has now been broadly enabled by the development of genetically-encoded biosensors (Figure 1). These proteins have enabled optical detection of a very broad diversity of signal transduction molecules and protein-protein interaction events in a large diversity of cell types, tissues and whole organisms (Lohse et al., 2008; Ohno et al., 2017; Greenwald et al., 2018; Ehrlich et al., 2019; Wright and Bouvier, 2021; Zhang et al., 2021b). The biosensor modality comprises protein(s) involved in a signal transduction event coupled to fluorescent and/or luminescent proteins that change in their optical properties when the signaling event occurs (e.g., elevation of cAMP, arrestin recruitment, receptor internalization) (Figure 1). The sensor can be delivered into cells via a suitable viral or plasmid vector or incorporated into the germline in genetically-manipulated animals. The sensors can be targeted to specific locations within the cell with the incorporation of localization sequences, enabling spatial resolution of signaling events (Vilardaga et al., 2014; Moore et al., 2016; Halls and Canals, 2018; Hilgendorf et al., 2019; Lobingier and Von Zastrow, 2019; Jullie et al., 2020; Zhang et al., 2021a). The time course of signaling is typically measured by default in these experiments, which has stimulated an explosion in the kinetic quantification of GPCR signaling. This has resulted in the discovery of new signaling mechanisms that modulate neuronal activity, for example persistent signaling by internalized GPCRs, and initiation of signaling at intracellular locations. These spatiotemporal mechanisms mediate GPCR function in pathophysiological conditions and are being targeted in the discovery and development of novel therapeutics (Vilardaga et al., 2014; Yarwood et al., 2017; Stoeber et al., 2018; Jimenez-Vargas et al., 2020).

Surprisingly, the wealth of time course data now being generated is rarely analyzed by curve fitting to extract kinetic signaling parameters. Instead, the time course data are typically represented in graphs and the insight is limited to qualitative interpretations derived from visual observation of the graphical data. Historically, curve fitting has transformed pharmacology and receptor research into rigorously quantitative disciplines (Kenakin, 2019). For example, the fitting of concentration-response data to the sigmoid curve equation using non-linear regression software enabled accurate and rapid quantification of potency and efficacy measurements (e.g., EC_50_ and E_max_). These measurements revealed the mechanisms of receptor function and provide the data that guides modern medicinal chemistry (Rang, 2006; Kenakin, 2019). This required two developments–equations that provide informative parameters such as EC_50_, E_max_, *K*_i_, and signal transduction parameters such as τ (Hill, 1909; Langmuir, 1918; Clark, 1926; Gaddum, 1937; Arunlakshana and Schild, 1959; Cheng and Prusoff, 1973; Black and Leff, 1983); and curve fitting software that can be easily used by scientists doing the research (Munson and Rodbard, 1980; Motulsky and Ransnas, 1987; Beck et al., 2004). Until recently such tools were not generally available for analyzing signaling kinetic data for GPCRs. To enable such analysis, we have introduced a data analysis framework for curve fitting to time course data for GPCR signaling. In this framework, data are fit to straightforward equations using familiar curve fitting software. The analysis provides fitted values of useful parameters, such as the initial rate of signaling and the rates of receptor desensitization and second messenger degradation. We have presented theoretical and practical studies of this new and evolving analysis paradigm (Hoare et al., 2018, 2020a,b; Hoare and Hughes, 2021) (see also https://youtu.be/_Pb7Sq6lZIY). Other straightforward approaches for curve fitting time course data have also been introduced recently (Zhu et al., 2019, 2020).

The goals of this study were to extend the practical application of signaling dynamic measurements and time course curve fitting to GPCR drug targets and receptor mechanisms relevant to the central nervous system. First, we introduce an updated and comprehensive collection of equations, provided as a plug-in for the popular program GraphPad Prism, to enable investigators to fit time course data for a variety of different experimental paradigms (see https://drive.google.com/drive/u/1/folders/1F5Qlyi30a3VNu9ZzCTKuTCDEmH6B4rdX). Second, we apply the kinetic signaling biosensor assays and data analysis to high-value CNS research questions, including the real-time quantitative determination of the signaling efficacy of opioid and cannabinoid receptors, and mechanisms of arrestin recruitment. This quantification of signaling kinetics provides quantitative insight into drug activity and receptor mechanisms and provides a framework for investigators to apply to their systems and questions of interest.

## Curve Fitting for Time Course Signaling Data

Recently, routine curve fitting methods have been introduced for analysis of time course data for GPCR signaling (Hoare et al., 2020b; Hoare and Hughes, 2021). Equations have been derived that describe the time course curve shapes, and the data are fit to these equations to estimate useful parameters such as rate constants and steady-state signaling levels. The shape of the curve and the equation used is dependent on the signal being measured. Some responses rise and then plateau at a steady-state level, whereas others rise to a peak and then decline. We recently published a survey of the time course curve shapes and discovered that four shapes/equations account for the large majority of GPCR signaling time course data (Hoare et al., 2020b). This limited number enables the curve fitting to be reduced to practice. Importantly, the curve shapes all emerge from a mechanistic foundation; when formulated mathematically the known mechanisms of GPCR signaling and regulation yield the four curve shape equations (Hoare et al., 2018, 2020b). GPCR signaling is regulated to prevent excessive stimulation of the cell, by the process of receptor desensitization which prevents further generation of the signal, and by degradation of the signal itself (for example, metabolism of second messengers) (Chang, 1968; Moore et al., 2007). The regulation mechanisms in operation in the cell control the shape of the time course (Hoare et al., 2020b).

**Figure 2:**
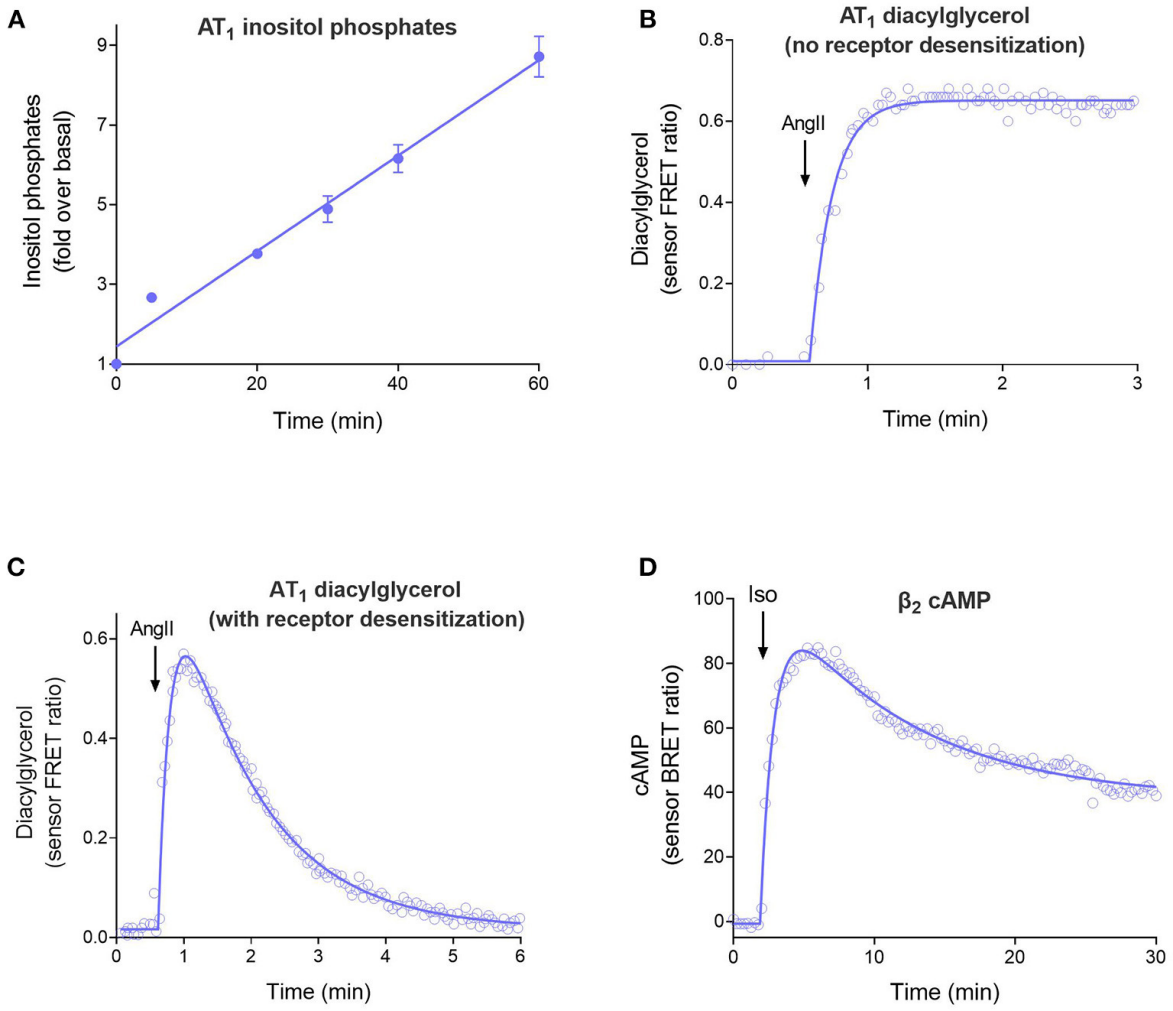
The four time course curve shapes for GPCR signaling. Almost all signaling time course data can be described by one of the four shapes shown here. The shape is determined by the regulation of signaling mechanisms in operation in the assay system (Hoare et al., 2020b). **(A)** Straight line time course, exemplified by inositol phosphates accumulation stimulated by angiotensinII (Ang II) via the AT_1_ angiotensin receptor (Kohout et al., 2001). Note there is no regulation of signaling in operation in the cells; receptor desensitization was blocked by the use of arrestin knock-out cells, and degradation of the inositol phosphates signal was blocked using LiCl. **(B)** Rise to steady-state curve, exemplified by AT_1_ receptor-stimulated diacylglycerol (DAG) production (Violin et al., 2006a). This curve results from a single regulation of signaling mechanism, in this case clearance of the DAG signal by DAG kinases. (Desensitization was blocked using a mutant receptor). **(C)** Rise and fall to baseline curve, exemplified by exemplified by AT_1_ receptor-stimulated DAG production via the wild-type receptor. This shape results when two regulation of signaling mechanisms are operative, in this case DAG degradation and receptor desensitization. **(D)** Rise and fall to steady-state curve, exemplified by β_2_ adrenoceptor-stimulated cAMP production (Thomsen et al., 2016). This mechanism results from more complex mechanisms, in this case likely involving receptor resensitization as well-desensitization (Hoare et al., 2020b).

The four curve shapes are shown in Figure 2 and are as follows:

1. Straight line (Figure 2A). The signal increases continuously over time at a constant rate. This time course occurs when there is no regulation of signaling and arises because the receptor continuously generates the signal. See Equation 1 in the Appendix in Supplementary Material.
2. Rise to steady-state curve (Figure 2B, Equation 2 in Appendix), also called the association exponential curve. The signal increases rapidly at first, then slows, then approaches a plateau at which the signal remains constant over time. This is a commonly-observed shape in GPCR second messenger assays and emerges because the signal becomes limited by a regulation of signaling mechanism (for example receptor desensitization or signal degradation). This shape arises when there is one predominant regulation mechanism.
3. The rise-and-fall to baseline curve (Figure 2C, Equation 3). The signal rises rapidly, then slows, then reaches a peak, following which the signal declines back down to the baseline level before initiation of the signal. This shape is observed in calcium mobilization assays, and in second messenger assays when blockers of metabolism of the messenger are excluded from the assay. Again, the shape is a manifestation of the regulation of signaling mechanisms. It arises when there are two mechanisms regulating the signal transduction pathway being measured (e.g., receptor desensitization and signal degradation).
4. The rise-and-fall to steady-state curve (Figure 2D, Equation 4). The signal rises rapidly, then slows, then reaches a peak, then declines. The signal then declines to a plateau level which is above the baseline but below the peak. This shape is a manifestation of more complex regulation mechanisms, including receptor resensitization, reformation of the signal after it has been degraded, and signaling by internalized receptors. These mechanisms have in common an initial burst of signaling, followed by processes that produce a steady-state of continuous signaling over time.

The equations defining these curve shapes are reasonably straightforward – investigators capable of performing concentration-response analysis should be able to perform time course signaling analysis with some training and experience. To aid investigators, in this study we introduce a plug-in for the program Prism (GraphPad Software Inc) comprising a suite of equations written in a common format, available at this location: https://drive.google.com/drive/folders/1F5Qlyi30a3VNu9ZzCTKuTCDEmH6B4rdX?usp=sharing. A guide for uploading the equations into Prism is provided in a file at the location above called “Guide for loading equations into Prism from a file”. We have also created a video training workshop, available here: https://youtu.be/_Pb7Sq6lZIY.

The suite of equations is comprehensive. It includes variants for downward signals (fall to steady-state curve, and fall and rise curves), to accommodate signals that decrease on receptor activation (e.g., inhibition of cAMP production by Gi-activated receptors). Variants also accommodate a baseline run-in period, where signal is measured before receptor is activated by application of the agonist. Finally, the equations are extended to allow for baseline drift, the slight change of baseline signal over time that can occur for biological or technical reasons (e.g., slight bleaching of fluorescent biosensors). A complete list of the equations together with illustrative graphs of the curve shapes, is in the Supplementary Material File, “Time course equation list.”

## Quantifying Signal Generation Using the Initial Rate of Signaling

The time course curve shapes arise from two biological processes. The first process to occur is the generation of the signal, where the agonist-occupied receptor generates the signal from precursors of the signal (for example, activated GTP-bound G-protein generated from inactive GDP-bound G-protein) (Gilman, 1987). The second process, which occurs after signal generation, is the regulation of signaling steps that act to dampen down or turn off the signal (for example, receptor desensitization and signal degradation) (Moore et al., 2007). One of the benefits of kinetic analysis, which is not possible with endpoint analysis, is that these processes can be separated and then quantified independently in the curve fitting analysis (Hoare et al., 2020b; Zhu et al., 2020).

The first process, the generation of the signal, is surprisingly easy to quantify. Signal generation can be quantified as the initial rate of signaling, analogous to the initial rate of enzyme activity. Traditional methods to measure the initial rate, involving manual assessments of which part of the curve is linear, are not suitable for the modern automated era of data analysis. Instead, we have developed a method that employs the curve fit parameter values to calculate the initial rate (Hoare et al., 2020b). In this method, the time course data are fit to the appropriate equation, then the fitted parameter values are entered into a formula that calculates the initial rate. These formulas are shown in Equations 5–8 in Appendix. To aid investigators, the Prism analysis we developed performs the initial rate calculation automatically as part of the analysis in most cases.

The rate of signal generation is highly useful because it represents the efficacy of the agonist for activating the receptor, unencumbered by regulation of signaling mechanisms (Hoare et al., 2020b). In pharmacology research, measuring receptor activation by drug molecules is of primary importance because it can translate to how well the drug will produce a therapeutic effect (Kenakin, 2009). Two targets in the CNS where this is of high importance are the μ-opioid receptor (Schmid et al., 2017; Gillis et al., 2020a; Pineyro and Nagi, 2021) and CB_1_ cannabinoid receptor (Banister and Connor, 2018a; Sachdev et al., 2019). Precisely defining receptor activation for these targets is important for defining therapeutic efficacy, and also for assessing the risk of adverse events. Consequently, in this study we quantified the rate of signal generation, using the initial rate of signaling, for these targets (see below).

The second process, the regulation of signaling steps, can also be quantified (Hoare et al., 2018, 2020b; Zhu et al., 2020). Macroscopically, the rate(s) of signal regulation can be quantified empirically as an overall rate constant or constants (Hoare et al., 2020b). From the rate constant value(s), the half-time(s) of the signal regulation step(s) can be determined. Microscopically, if the mechanisms of signal regulation are known, then it is also possible to quantify the rates of these processes, for example the rates of receptor desensitization and signal degradation (Hoare et al., 2020b). This aspect of the kinetic analysis is applied here to arrestin recruitment to a series of GPCRs under a variety of conditions.

## Quantifying Signal Generation by the μ-Opioid Receptor

### Introduction

Drugs that activate the μ-opioid receptor (MOR) are highly effective analgesics, the classic example being morphine. Analgesia is achieved by activation of this receptor at multiple CNS sites (Pasternak, 1993). However, side effects result from MOR activation at other locations, including respiratory depression (which can be fatal) and constipation (Pasternak, 1993; Gillis et al., 2020b). In addition, tolerance and dependence can result on repeated dosing of MOR agonists (Johnson et al., 2005; Morgan and Christie, 2011), which has resulted in widespread opioid use disorder, contributing to the opioid crisis which led to 70,000 fatalities in the United States in 2019 (Mattson et al., 2021). Accurately quantifying the extent to which MOR agonists activate the receptor is essential for understanding the liabilities of current mediations and in the optimization of safer new therapeutics targeting this receptor (Thompson et al., 2016; Schmid et al., 2017; Gillis et al., 2020c; Hill and Canals, 2021; Pineyro and Nagi, 2021). This quantification is central to the two approaches currently being advocated, which are: (1) Partial agonism, the development of ligands that only partially activate the receptor, sufficient to achieve analgesia but low enough to minimize side effects (Gillis et al., 2020a). (2) Biased agonism, the development of ligands that are biased for activating G-protein vs. recruiting arrestin (Schmid et al., 2017).

Kinetic analysis enables accurate quantification of the strength of the signal generation event stimulated by agonists (Hoare et al., 2020a,b; Yang et al., 2021). Notably, the analysis allows the quantification of the signal generation event to be separated from the regulation of signaling events, important because differential regulation can result in differences in apparent efficacy of ligands (Klein Herenbrink et al., 2016). In this study we used high-resolution kinetic data obtained using biosensors to quantify the rate of signal generation by agonist-bound MORs. We measured the G-protein pathway by measuring cAMP using the Green Downward cADDis sensor (Tewson et al., 2016, 2018). The MOR receptor couples to Gi, which inhibits adenylyl cyclase, and this pathway can be quantified by measuring the decrease of cAMP generation after application of the MOR agonist. We measured the arrestin pathway using a fluorescent arrestin biosensor (Hoare et al., 2020a). Both biosensors are direct fluorescent biosensors in which the signaling event (binding of cAMP, or receptor-arrestin interaction) results in a change in fluorescence intensity which can be measured in a plate reader (Figure 1). These sensors are ideal for kinetic measurements because they are bright, enabling short read times and so a large number of time points, and because the signal is not susceptible to rapid decay effects such as decay of light-generating substrates in BRET assays and photobleaching in FRET assays. The receptor and the biosensor were expressed together in HEK293 cells using the BacMam expression system (Kost et al., 2007). Signaling was quantified as the change of fluorescence intensity, measured using the Hamamatsu FDSS/μCell Functional Drug Screening System (Hamamatsu Photonics), which enabled simultaneous scanning of an entire 384-well-plate. For the cAMP assay, inhibition of forskolin-stimulated cAMP was measured. Forskolin was applied 50 min before the MOR agonist, which was long enough for the forskolin response to reach steady-state [see Figure 5 of Hoare and Hughes (2021)]. This enables the kinetics of the MOR agonist activity to be assessed independently of the kinetics of forskolin activity.

### Signal Generation Rate of cAMP Inhibition by μ Opioid Receptor Agonists

**Figure 3:**
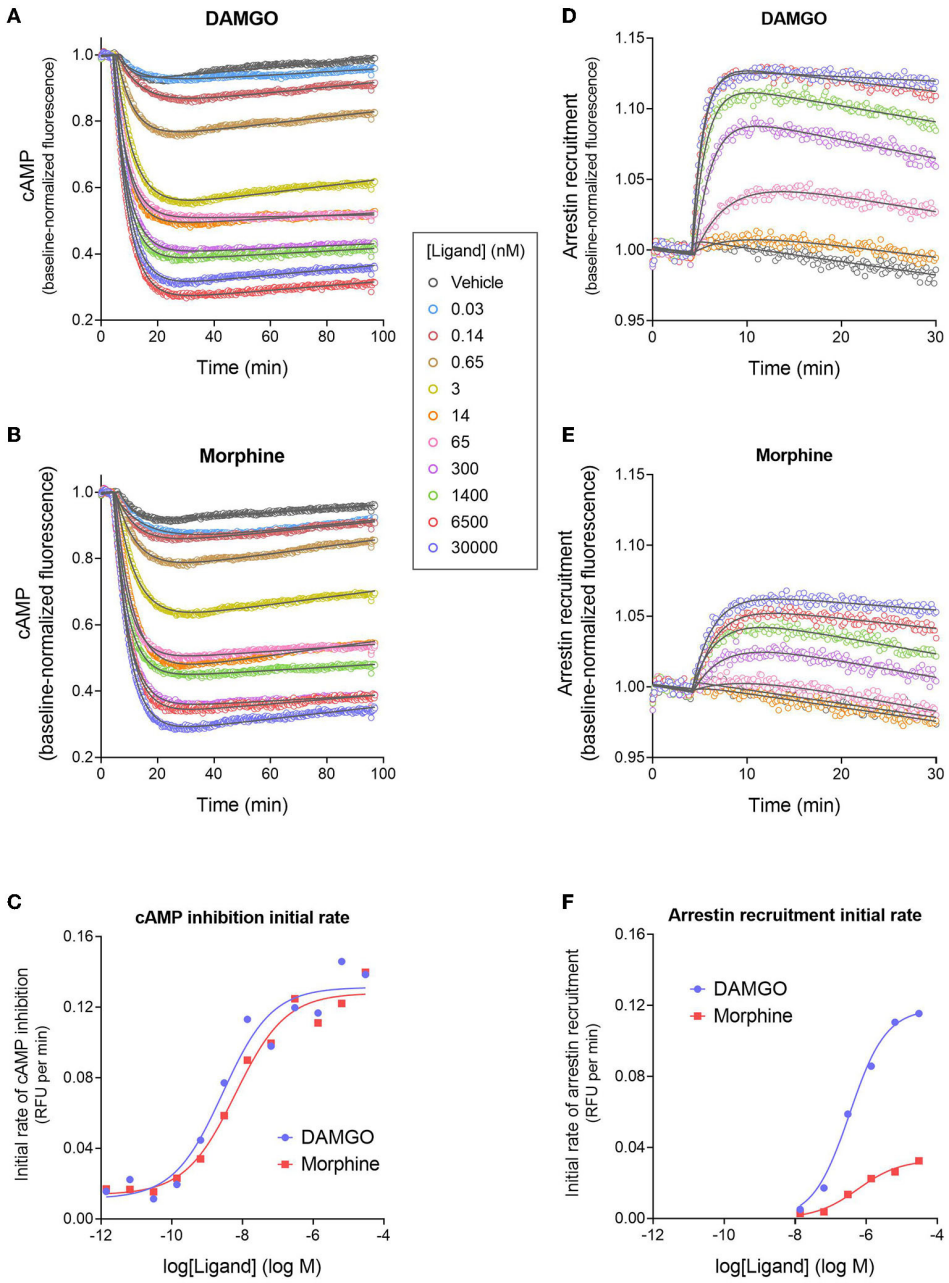
Signaling kinetics and signal generation rate of the μ opioid receptor for inhibition of cAMP production and stimulation of arrestin recruitment. The signals were measured repeatedly over time using fluorescent biosensors in HEK293 cells. **(A,B)** Time course of cAMP inhibition by DAMGO **(A)** and morphine **(B)**. Data were fit to the “Baseline then fall to steady state with drift” equation in GraphPad Prism (gray lines). From the fitted parameter values, the initial rate of cAMP inhibition was calculated using the formula Initial rate = SteadyState × K. In **(C)**, the initial rate is plotted vs. the DAMGO or morphine concentration and the data fit to a sigmoid curve concentration response equation (Motulsky, 2019). From this fit, the initial rate of cAMP inhibition by the agonist-occupied receptor (IR_max_) was determined, as the Span value (Top minus Bottom). Note morphine is a full agonist (IR_max_ of 95 % of DAMGO IR_max_). **(D,E)** Time course of arrestin recruitment to the MOR stimulated by DAMGO **(D)** and morphine **(E)**. Data were fit to fit to the “Baseline then rise to steady state with drift” equation in GraphPad Prism (gray lines). The initial rate of arrestin recruitment was calculated from the fit parameters using the formula Initial rate = SteadyState × K. **(F)** Initial rate of arrestin recruitment vs. agonist concentration. The IR_max_ for the agonists was determined as the Span value of the sigmoid curve fit. Note morphine is a partial agonist (IR_max_ of 28 % of DAMGO IR_max_). Data are from a representative experiment. Data points in **(A,B,D,E)** are the mean of two technical replicates (note, error bars have been excluded for clarity).

Figures 3A,B shows the time course of inhibition of cAMP production stimulated by forskolin after application of MOR agonists DAMGO (the standard reference agonist) and the analgesic drug morphine. After application of the agonist, there was a rapid reduction of the cAMP level over the first few minutes, which was presumably a result of Gi activation by the receptor. The effect slowed down over time and the inhibition reached a lower plateau, described by a fall to steady-state curve. However, this plateau drifted over time, evident by the slightly increasing signal over the later time points (Figures 3A,B). This baseline drift is probably a result of slight photobleaching of the biosensor since a high scan frequency (2 sec) was applied for a prolonged period of time (90 min), and because it was evident in vehicle-treated cells.

Next we analyzed the cAMP inhibition time course data by curve fitting (Figures 3A,B). The curve shape can be analyzed by incorporating baseline drift into the curve fitting analysis (Hoare et al., 2020a; Hoare and Hughes, 2021) and we have developed equations to handle this scenario. Data were analyzed using a fall to plateau equation with baseline drift, as described in Materials and Methods. This was performed with GraphPad Prism using the custom equation “Baseline then fall to steady state with drift” available in the file “[Pharmechanics] Fall to steady state equations” at https://drive.google.com/drive/folders/1F5Qlyi30a3VNu9ZzCTKuTCDEmH6B4rdX?usp=sharing. The data were fit well by the equation (*R*^2^ values of 0.967–0.999 for the data in Figures 3A,B). The parameters fitted by this equation are the steady-state level of cAMP inhibition (“SteadyState”), which is the baseline before application of the agonist minus the lower plateau; the rate constant (K), which defines the timeframe over which the cAMP inhibition occurs and is related to the half-time of the response (t_1/2_ = 0.693/K); and the drift factor, which defines the rate of drift of the response over time. The Supplementary Material File “Mu opioid time course curve fit results” shows the SteadyState, K and t_1/2_ values from the analysis. From these results we can determine the dependence on the agonist concentration of the fitted values. Increasing the agonist concentration increased the steady-state level of cAMP inhibition but did not appreciably affect the t_1/2_. This is also evident by visual inspection of Figures 3A,B. The mechanisms underlying the dependence of the SteadyState and K values on agonist concentration can be complex, as described previously (Hoare et al., 2018).

We used these data to determine the rate of signal-generation by the agonist-bound receptor, which is the initial rate of signaling by the receptor when it is fully occupied by the agonist; this is the efficacy of the agonist-occupied receptor for generating the signal. This parameter is termed IR_max_ (Hoare et al., 2020b), meaning the initial rate at a maximally-effective concentration of agonist. First, we calculated the initial rate for all the agonist concentrations. This was done by entering the curve fit parameters into a simple formula, which is: Initial rate = SteadyState × K (Hoare et al., 2020b; see Supplementary Material File “Mu opioid time course curve fit results” for values). These initial rate values were then plotted vs. the agonist concentration, as shown in Figure 3C. This plot shows that as the agonist concentration increases, the initial rate of signal generation increases, and this is because the number of agonist-bound receptors is increasing. These data were analyzed to determine IR_max_. This was done by fitting the initial rate vs. concentration data to a standard sigmoid curve equation (Motulsky, 2019) (see Materials and Methods). The IR_max_ is the maximal span of the curve, which is the Top minus the Bottom (Note the Bottom value is slightly above zero and this is because of a small injection artifact, evident from the vehicle data in Figures 3A,B). This analysis yielded an IR_max_ value of 0.120 relative fluorescence units (RFU) per min for DAMGO and 0.114 RFU per min for morphine for the representative experiment in Figure 3. It is convenient to express the IR_max_ as a percentage of that for a reference agonist, as is done for more traditional measures of agonist efficacy. This gave normalized IR_max_ values of 100 % for DAMGO and 103 % for morphine (mean of two independent experiments, Table 1). This result demonstrates that morphine is a full agonist for generating the inhibition of cAMP signal via the MOR in this cell system.

**Table 1:** μ opioid signal generation rate values for inhibition of cAMP production and stimulation of arrestin recruitment.

**Agonist**	**cAMP IR_**max**_ (% DAMGO)**	**Arrestin IR_**max**_ (% DAMGO)**
DAMGO	100	100
Met-enkephalin	90 ± 10	97 ± 2
Endomorphin-2	96 ± 1	71 ± 2
Morphine	103 ± 7	33 ± 4
Hydromorphone	94 ± 5	22 ± 3
Oxymorphone	106 ± 16	26 ± 6
Fentanyl	107 ± 16	46 ± 4
Buprenorphine	78 ± 4	ND

**Figure 4:**
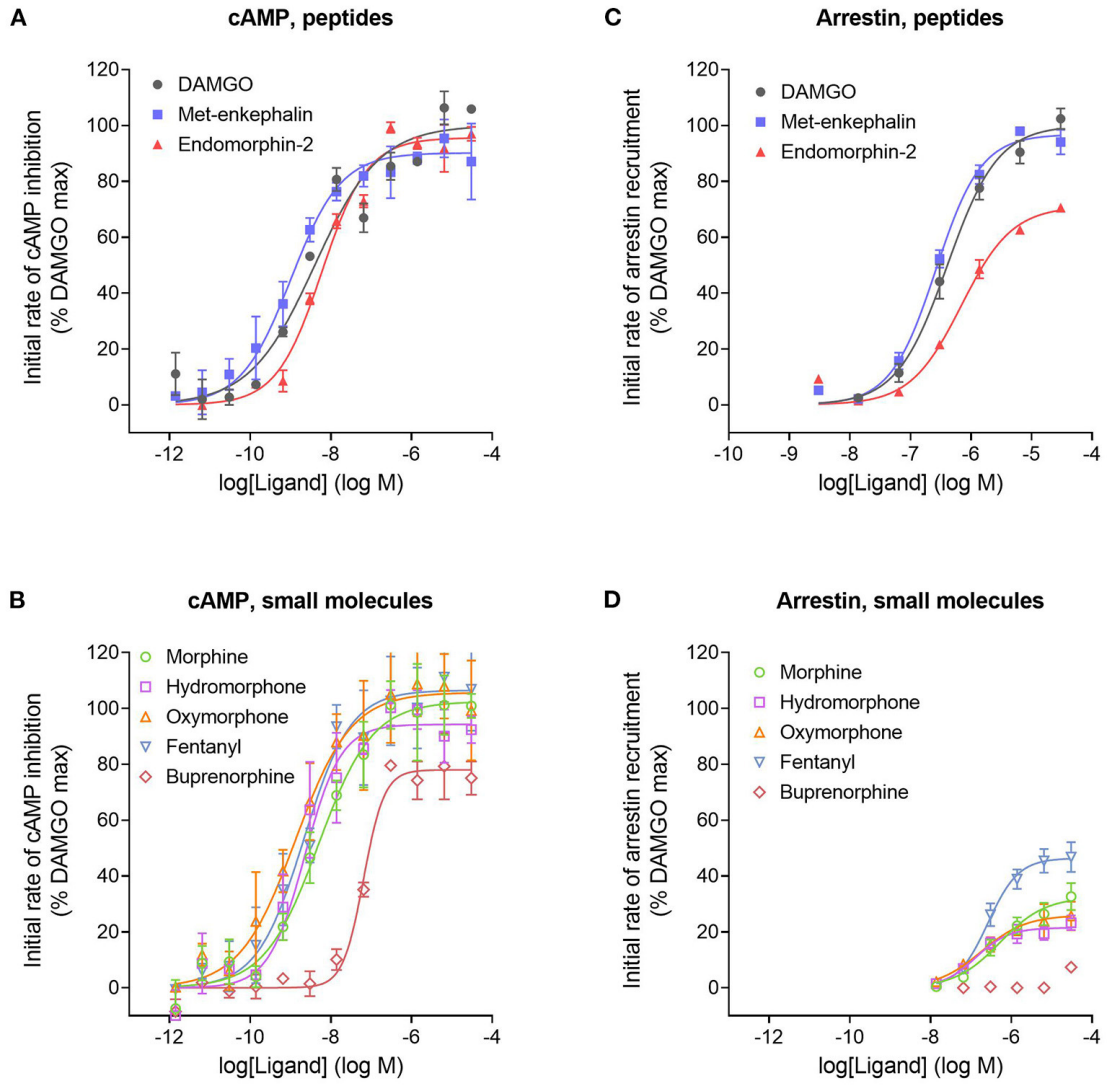
Initial rate of signal generation by the μ opioid receptor in response to endogenous peptide ligands **(A,C)** and small molecule therapeutics **(B,D)**. The initial rate of cAMP inhibition **(A,B)** and arrestin recruitment **(C,D)** was calculated from the time course data curve fit parameters (Figure 3, Supplementary Figures 1, 2). Data points are the mean ± SEM from two separate experiments. The curves were generated using the sigmoid curve equation (Motulsky, 2019), defined by the average curve fit values from the two experiments from Supplementary Table 1. Note that the small molecules are all partial agonists for generating arrestin recruitment.

*The signal generation rate of the agonist-occupied receptor, which is the initial rate of signaling at maximally-effective agonist concentrations (IR_max_), was quantified as the maximum span of the initial rate concentration response curve (Figure 4), normalized to that of DAMGO. Values are the mean ± SEM of values from two independent experiments. ND, not detected*.

We next tested a panel of MOR agonists in the cAMP inhibition assay (Figures 4A,B). The time course curve data are shown in Supplementary Figure 1 and the curve fit results in the Supplementary Material File “Mu opioid time course curve fit results.” The time course curve shape for all ligands was the same as that for DAMGO and morphine (fall to steady-state with baseline drift). The initial rate of cAMP inhibition was determined as described above and the values were normalized as a percentage of the maximal initial rate (IR_max_) of DAMGO, run as a control in each experiment. These normalized initial rate values are shown in Figures 4A,B. From these data the IR_max_ of the ligands, representing the signal generation rate by the agonist-bound receptor, was calculated as the Span of the sigmoid equation fit (Table 1). Seven of the eight agonists tested were full agonists for generation of cAMP inhibition, with IR_max_ values close to 100% (Table 1). These were the peptide agonists DAMGO, met-enkephalin and endomorphin-2, and the small molecule analgesic drugs morphine, hydromorphone, oxymorphone and fentanyl (Table 1). One of the agonists was a partial agonist for generation of cAMP inhibition; for buprenorphine the IR_max_ was 78%.

### Signal Generation Rate of Arrestin Recruitment by μ Opioid Receptor Agonists

We next measured the signal generation rate for arrestin recruitment to the MOR, using the fluorescent biosensor technology. We employed a fluorescent arrestin-3 (β-arrestin 2) biosensor described previously (Hoare et al., 2020a). This direct fluorescent sensor comprises arrestin-3 coupled to mNeonGreen (Figure 1C) and interaction with the receptor results in a change in fluorescence intensity that can be recorded in a plate reader. The time course of arrestin recruitment following application of the agonist is shown in Figures 3D,E, for DAMGO and morphine. There was a rapid initial rise phase occurring over the first few minutes, then the signal approached a plateau. This is represented by the rise to steady-state curve and equation. However, like in the cAMP assay, there was slight baseline drift, evident from the decline of the signal over time at the later time points (Figures 3D,E). The data were fit to an equation that describes these features, the “Baseline then rise to steady state with drift” equation, using GraphPad Prism, as described in Materials and Methods. The data were fit well by the equation (*R*^2^-values of 0.959–0.996 for the data in Figures 3D,E). The fitted parameter values are SteadyState (the steady-state level of arrestin recruitment), the rate constant K (from which the half-time can be calculated as 0.693 / K), and the drift factor. Increasing the agonist concentration increases the steady-state level of arrestin recruitment, and decreases the half-time (Figures 3D,E; see Supplementary Material File “Mu opioid time course curve fit results”). However, a difference between DAMGO and morphine is evident. Morphine is less effective for recruiting arrestin. The maximal steady-state level is less, and the half-time is greater (Figures 3D,E; see Supplementary Material File “Mu opioid time course curve fit results”).

Now we determined the signal generation rate for recruitment of arrestin by the agonist-occupied receptor (IR_max_). This was done as described above for the cAMP response. First, the initial rate for each concentration of agonist was calculated using the formula Initial rate = SteadyState × K (Hoare et al., 2020b; see Supplementary Material File “Mu opioid time course curve fit results” for values). Next the initial rate values were plotted against the agonist concentration (Figure 3F) and the data analyzed using a sigmoid curve equation (Motulsky, 2019). The IR_max_ was calculated as the Span value from this fit. It is immediately obvious from inspection of Figure 3F that the IR_max_ of morphine is considerably less than that of the reference agonist DAMGO (IR_max_ values of 0.0330 and 0.118 RFU per min, respectively). The mean normalized IR_max_ values, relative to DAMGO, are 33% for morphine and 100% for DAMGO (Table 1). This result demonstrates morphine is a partial agonist for generating recruitment of arrestin.

We next tested the panel of MOR agonists in this assay to quantify the generation of arrestin recruitment (time course data shown in Supplementary Figure 2, note data for all agonists were fit well by the rise to steady-state with drift equation). The normalized initial rate values are shown in Figures 4C,D and the IR_max_ values in Table 1. It is clear from these data that the small molecule analgesic drugs are all partial agonists for generating arrestin recruitment (morphine, hydromorphone, oxymorphone and fentanyl, IR_max_ ranging from 22 to 46%). Notably, buprenorphine did not detectably recruit arrestin (Figure 4D, Supplementary Figure 2F). The peptide agonist met-enkephalin was a full agonist relative to DAMGO (IR_max_ of 97 %), and the second peptide agonist tested, endomorphin-2, was a partial agonist (IR_max_ of 71 %).

### Comparison With Other Methods

Precisely quantifying ligand efficacy for signaling is critically important for developing next-generation analgesics targeting the μ opioid receptor, whether this is based on the partial agonist hypothesis (Gillis et al., 2020a) or the G-protein over arrestin bias hypothesis (Schmid et al., 2017). The IR_max_ signal generation rate measurement developed here is one of many scales of signaling strength employed, including E_max_, and transduction coefficients such as τ and E_max_/EC_50_ (Mcpherson et al., 2010; Thompson et al., 2016; Schmid et al., 2017; Ehrlich et al., 2019; Gillis et al., 2020a; Uprety et al., 2021). The signal generation rate measurement has benefits and limitations. It quantifies signal generation before the signal is modified by regulation of signaling mechanisms (Hoare et al., 2020b). Consequently, this measurement reflects the activating conformation of the receptor before regulation of signaling, providing an assessment of molecular efficacy of the agonist for activating the receptor. In addition, using the signal generation rate avoids the time-dependence of agonist efficacy measurements that can emerge in endpoint assays (Klein Herenbrink et al., 2016; Thompson et al., 2016; Zhu et al., 2019; Hoare et al., 2020b). This time dependence is likely a result of regulation of signaling mechanisms that are dependent on the agonist, such as receptor desensitization and internalization (Zhu et al., 2019; Hoare et al., 2020b). However, the signal generation rate does not provide an estimate of tonic signaling that would be important for translation to *in vivo* models for agonists that produce sustained signaling. For this application, the time course method described here can still be applied – the relevant parameter from the curve fitting is SteadyState, the signal level at the plateau.

A major limitation of the signal generation rate measurement is that it can be difficult to incorporate the potency or affinity of the agonist for the receptor for rapid responses. This is because the method assumes the rate limiting step is generation of the signal by the agonist-occupied receptor. This assumption might be infringed at low concentrations of agonist needed to define the EC_50_, where the rate limiting step instead can be the binding of the agonist to the receptor (Hoare et al., 2018). This limitation profoundly distorts measurements when the response is very rapid [e.g., intracellular Ca^2+^ mobilization (Charlton and Vauquelin, 2010)], but is likely less of an issue for more slowly-generated signals (Bdioui et al., 2018; Hoare et al., 2018, 2020b). A second limitation is that receptor reserve / signal amplification is not presently incorporated into the signal generation rate measurement. Consequently, caution is required when using the signal generation rate in assessing biased agonism when one of the pathways is more amplified than the other, as is likely the case here, where inhibition of cAMP production is likely more amplified than arrestin recruitment.

Broadly, the signal generation rate IR_max_ results were in agreement with traditional measures of the maximal response (These traditional measures are usually single time point measurements, typically at later time points when the response is approaching or has reached steady-state). Specifically, for inhibition of cAMP the small molecule analgesics were full agonists, with the exception of buprenorphine which was a partial agonist, albeit with an efficacy >50% (Table 1), and these findings are in agreement with previous studies (Zaki et al., 2000; Knapman et al., 2014; Schmid et al., 2017; Gillis et al., 2020a). This agreement between the initial rate and steady-state methods for cAMP inhibition likely results because the initial rate is defined largely by the magnitude of the SteadyState parameter, with the rate parameter K being similar for the different agonists, as described in Hoare et al. (2018; see Supplementary Material File, “Mu opioid time course curve fit results” for SteadyState and K values). For arrestin recruitment, the rank order of efficacy quantified using IR_max_ was similar to that of E_max_ measured in previous studies at a single time point, specifically DAMGO = met-enkephalin > endomorphin-2 > fentanyl > morphine > buprenorphine (Mcpherson et al., 2010; Rivero et al., 2012; Thompson et al., 2016; Schmid et al., 2017; Gillis et al., 2020a). The absolute values in the literature can vary widely. For example, the E_max_ for morphine varies from 15% (Mcpherson et al., 2010) to 72% (Thompson et al., 2016). This variability might be in part due to the time point used to make the measurement; in arrestin assays, the E_max_ of agonists can increase at later time points (Hoare et al., 2020a). This results from both the rate constant K and the SteadyState value being dependent on the concentration of the agonist (Hoare et al., 2020a), as was observed in this study (see Supplementary Material File, “Mu opioid time course curve fit results”). The signal generation rate method avoids this complication. Overall, the signal generation rate for arrestin recruitment described in this study provides one of the most sensitive and unambiguous assessments of agonist efficacy at the μ-opioid receptor, because it is not susceptible to amplification effects, is not time dependent, and is conceptually straightforward.

## Illicit Synthetic Cannabinoid Signal Generation Rate Via the CB_1_ Receptor

### Introduction

The CB_1_ receptor is the primary site of action of the natural cannabinoid Δ^9^-tetrahydrocannibinol (THC), the main psychoactive ingredient of cannabis (Paton and Pertwee, 1973). In recent times, synthetic cannabinoid receptor agonists (SCRAs) have been developed for research purposes that have subsequently been diverted and modified by illicit laboratories for recreational use (Bretteville-Jensen et al., 2013; Banister and Connor, 2018a,b). Unlike THC, some of these compounds have been associated with severe toxicological events, including seizures, cardiotoxicity, psychosis, hypothermia, and kidney injury, resulting in hundreds of hospitalizations and dozens of fatalities (Trecki et al., 2015; Adams et al., 2017). This increased morbidity and mortality of some of the SCRAs is correlated with a stronger efficacy for activation of the CB_1_ receptor (Wiley et al., 2015; Banister et al., 2016; Cannaert et al., 2016; Hess et al., 2016; Costain et al., 2018; Gamage et al., 2018; Grafinger et al., 2021a,b). For example, the SCRA 5F-MDMB-PICA was recently shown to activate the CB_1_ receptor with an efficacy 260-fold higher than that of THC, demonstrated using the operational model of agonism and varying levels of receptor expression (Sachdev et al., 2019).

### Rate of Signal Generation via the CB_1_ Receptor

We examined whether this difference of signaling strength was also evident in terms of the rate of signal generation. For this purpose, we utilized time course data for activation of CB_1_ receptor signaling from a recent study which included extensive characterization of SCRA pharmacological efficacy at the CB_1_ receptor (Sachdev et al., 2019). The response measured was hyperpolarization of AtT-20 cells expressing the human CB_1_ receptor and this was detected using a fluorescent membrane potential-sensing dye. The change of fluorescence was directly related to the change of membrane potential resulting from CB_1_ receptor activation, followed by release of G-protein βγ subunits, and subsequent downstream activation of endogenous GIRK channels (Mackie et al., 1995; Garcia et al., 1998; Sachdev et al., 2019). This assay has a wide dynamic range for detecting differences of ligand efficacy because it is not highly amplified; four G-protein βγ subunits are likely required to fully activate the GIRK channel (Whorton and Mackinnon, 2013). In addition, the number of CB_1_ receptors in the cells was relatively low because an irreversible antagonist was used to reduce the number of receptors accessible to the agonist ligands (Sachdev et al., 2019).

**Figure 5:**
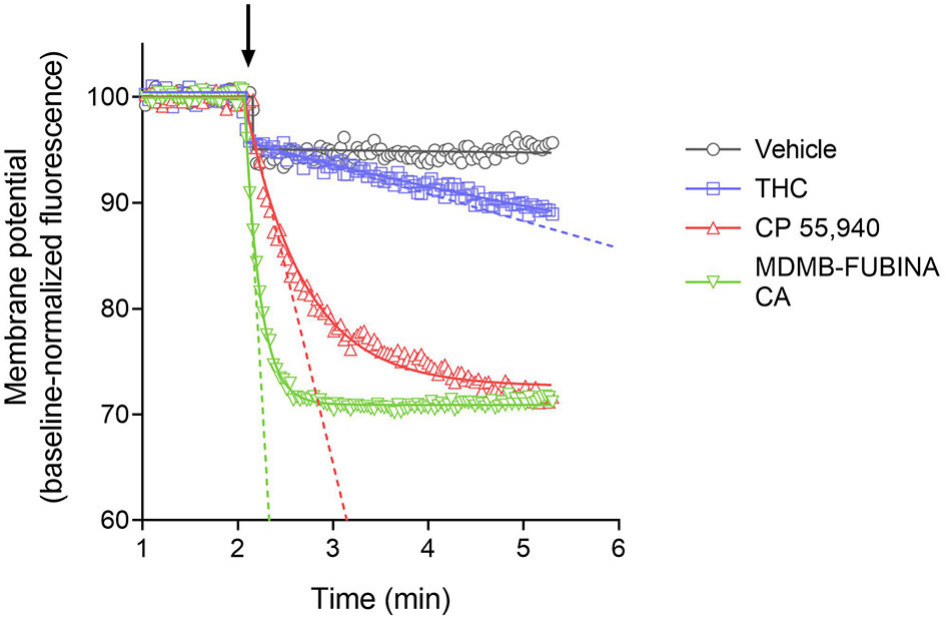
Signaling kinetics of CB_1_ receptor-mediated membrane potential reduction stimulated by CB_1_ agonists. Membrane potential was measured using a fluorescent dye in AtT20 cells and data normalized to the baseline signal before application of agonist or vehicle (indicated by arrow). Data were analyzed by curve fitting to fall to steady-state equations as described in Materials and Methods. A maximally-stimulating concentration was used (Table 2). THC produced a slow, small reduction of membrane potential, detectable beyond the small injection artifact reduction evident in the vehicle condition. The synthetic agonists CP 55,940 and MDMB-FUBINACA produced a more rapid and larger reduction (See the Supplementary Material File “CB_1_ hyperpolarization time course fit results” for curve fit parameter results). The initial rate of signal generation by the agonist-occupied receptor (IR_max_) was calculated from the curve fit parameters and this rate is indicated by the dashed line on the graph. Note the initial rate is much faster for CP 55,940 and MDMB-FUBINACA compared with THC. Data points are the mean of two technical replicates and data are from representative experiments.

We quantified the kinetics of ten CB_1_ agonists in this assay using a maximally-effective concentration of agonist (Table 2). This enabled us to quantify the IR_max_ of the ligands. Figure 5 shows the time course data for the change of membrane potential following application of three of the agonists or the vehicle. Note that in this assay there was a small injection artifact that produced an immediate reduction of the signal, evident in the vehicle and THC condition; this was taken into account in the curve fitting and data analysis (see Materials and Methods). THC produced a slow, small reduction of the membrane potential. By contrast, the synthetic ligands CP 55,940 and MDMB-FUBINACA produced a much more rapid and larger reduction of membrane potential (Figure 5, Table 2). The data were fit to the fall to steady-state equation to quantify SteadyState (the final reduction of the response) and the rate constant K (mean values shown in Table 2). The SteadyState value was corrected for the small signal deflection caused by the injection artifact as described in Materials and Methods. The fitted values for each experiment are provided in the Supplementary Material File, “CB_1_ hyperpolarization time course fit results” and the time course curve fit for each ligand shown in Supplementary Figure 3.

**Table 2:** THC and SRCA signal generation rate via the CB_1_ receptor.

**Agonist**	**IR_**max**_ (% per min)**	**IR_**max**_/THC IR_**max**_**	**SteadyState (% reduction)**	**K (min^**−1**^)**	**Concentration (μM)**
THC	3.0 ± 0.4	1.0	15 ± 3	0.29 ± 0.09	10
CP 55,940	48 ± 7^*^	16	27 ± 1	1.8 ± 0.2	30
JWH-018	27 ± 4 ^NS^	9.0	20 ± 3	1.3 ± 0.07	10
AM-2201	59 ± 7^***^	20	21 ± 3	3.0 ± 0.3	10
XLR-11	60 ± 10^**^	20	24 ± 3	2.6 ± 0.2	10
5F-PB-22	100 ± 10^***^	35	22 ± 2	4.8 ± 0.2	1
MDMB-CHMICA	140 ± 12^***^	45	28 ± 3	4.9 ± 0.3	1
MDMB-FUBINACA	120 ± 10^***^	41	27 ± 3	4.7 ± 0.5	1
5F-MDMB-PICA	150 ± 10^***^	49	30 ± 2	4.9 ± 0.3	1
CUMYL-4CN-BINACA	110 ± 10^***^	36	25 ± 1	4.2 ± 0.5	10

*The IR_max_ was quantified for reduction of membrane potential stimulated by a maximally-effective concentration of the agonist. Also shown are the SteadyState and K rate constant values from the curve fitting to the time course data, used to calculate the IR_max_ using the equation, Initial rate = SteadyState × K. The SteadyState value was corrected for the injection artifact which resulted in a slight, immediate drop of the signal (see Materials and Methods). Values are mean ± SEM from 5 to 9 independent experiments. Differences of IR_max_ between test compounds and THC were tested statistically by single-factor ANOVA followed by the Dunnett multiple comparison test, comparing each compound with THC (*

* *P < 0.05*;

** *P < 0.01*;

*** *P < 0.001*;

NS *, not significant, P > 0.05)*.

**Figure 6:**
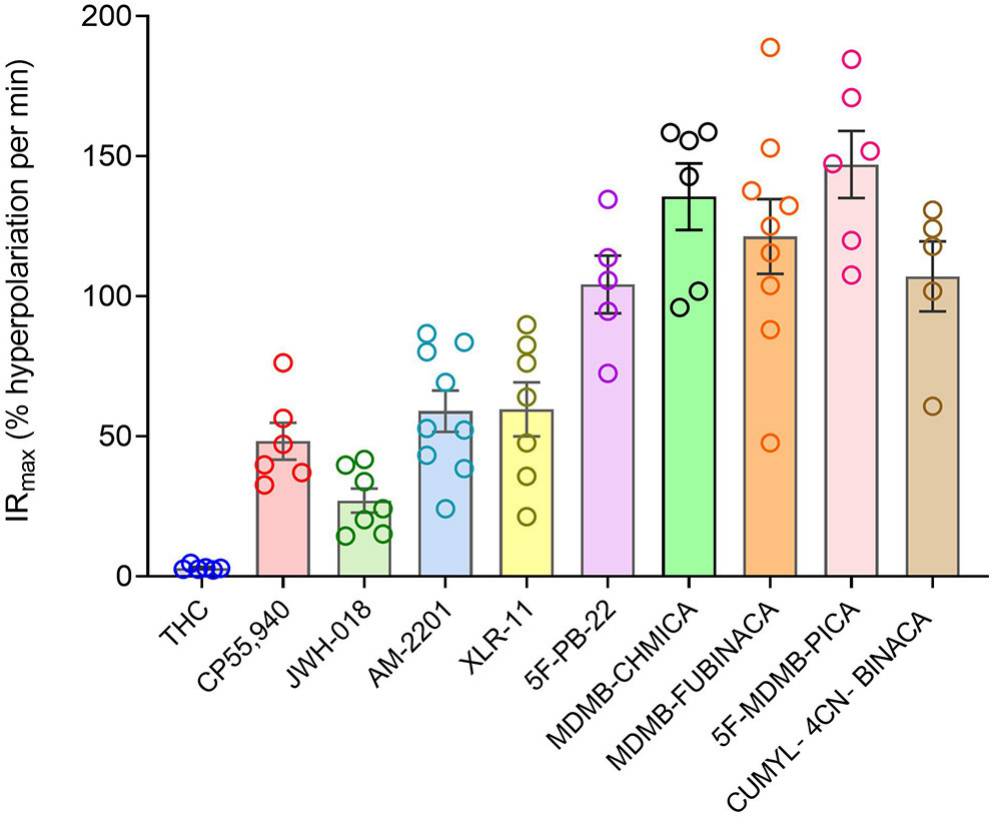
Signal generation rate IR_max_ values of CB_1_ receptor agonists. The IR_max_ was calculated as the initial rate of membrane potential reduction at a maximally-stimulating concentration of the agonists (10 μM), as described in Materials and Methods. Note the signal generation rate of SRCAs and the synthetic agonist CP 55,940 is much higher than that of the natural cannabinoid THC. IR_max_ values and statistical analysis are in Table 2.

Now we examined the signal generation rate by the agonist-occupied receptor. The IR_max_ value for each ligand was calculated by multiplying the corrected SteadyState value by the K value. The IR_max_ values are provided in Table 2 and shown in Figure 6. Clearly there was a very large difference of the signal generation rate between THC and the SCRAs. The IR_max_ value for THC was 3.0 % reduction of membrane potential per minute, whereas the values for the SCRAs were much higher. For example, for MDMB-FUBINACA the IR_max_ value was 40 times higher, at 120% reduction per minute. In all cases except JWH-018, the IR_max_ value was significantly different from that for THC (Table 2). This difference of IR_max_ value is clearly evident when the initial rate is plotted on the time course graph, as indicated by the dashed lines in Figure 5. Overall, the signal generation rate for SCRAs ranged from 9-fold to 49-fold higher than that of THC (Table 2). This result supports the hypothesis that the SCRAs more strongly activate CB_1_ receptor signaling. It seems probable that this difference contributes to the more severe CB_1_-mediated toxicology of SCRAs compared with THC. The difference between THC and MDMB-FUBINACA can be rationalized by differences of structure of the agonist-CB_1_ receptor complex; MDMB-FUBINACA demonstrated a “toggle twin switch” interaction that THC did not (Krishna Kumar et al., 2019). The present results suggest the different activate state of the receptor when bound by MDMB-FUBINACA accelerates activation of the receptor and subsequent signaling.

## Quantitative Mechanistic Analysis of Arrestin Recruitment Waveforms

### Introduction

**Figure 7:**
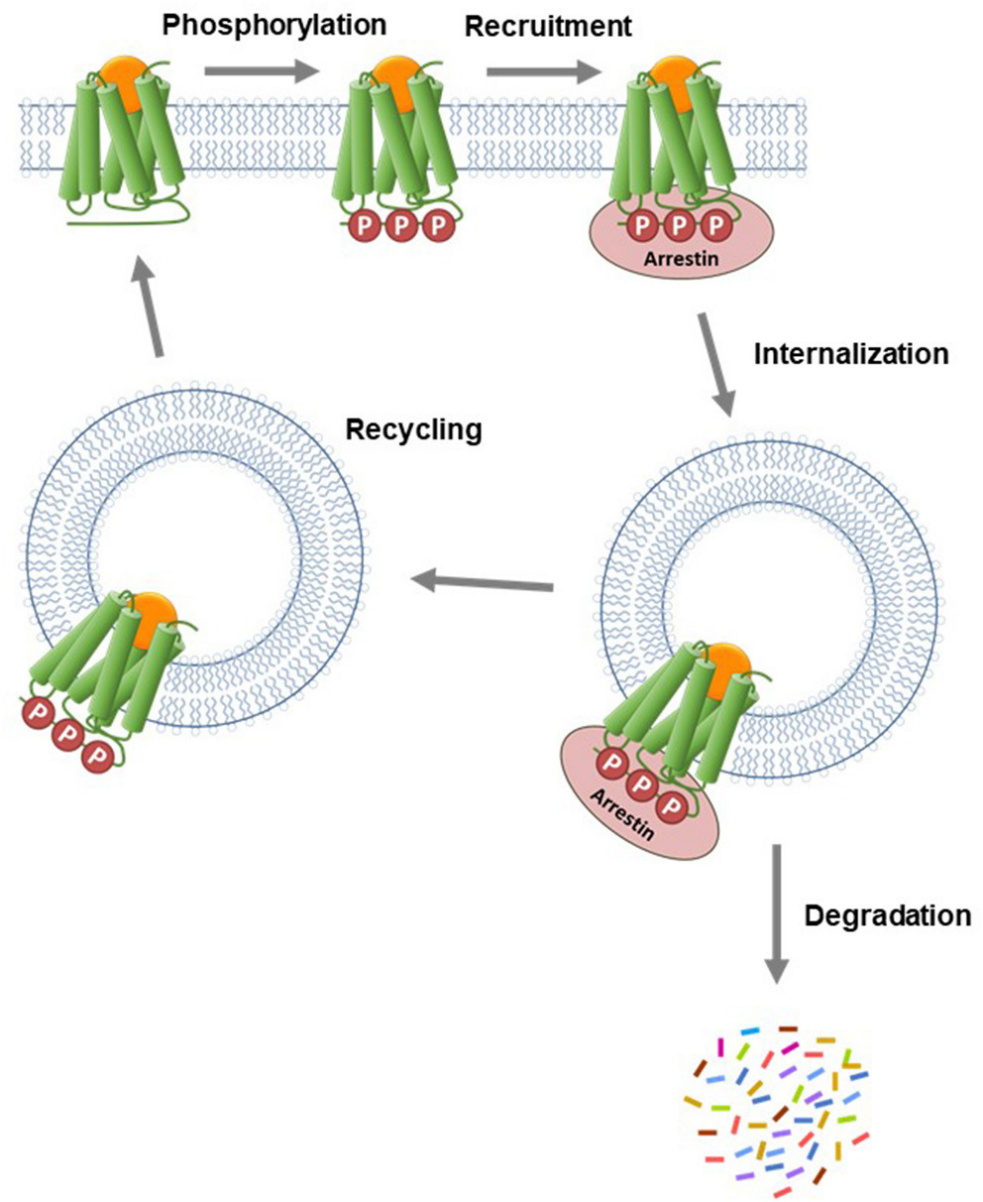
Canonical mechanism of arrestin recruitment and subsequent regulation of signaling events. The agonist-activated GPCR is phosphorylated by kinase enzymes on intracellular regions. The phosphorylated GPCR then recruits arrestin. This step blocks G-protein interaction and subsequent signaling and is the canonical mechanism of receptor desensitization. The receptor-arrestin complex is then internalized into endosomes via clathrin-coated pits. Following internalization, the receptor is trafficked via two primary pathways. Either the receptor is transported to lysosomes where it is degraded or it is recycled to the plasma membrane where it can contribute again to G-protein signaling.

The arrestin proteins perform multiple functions in regulating and mediating GPCR signaling. For most GPCRs, arrestins mediate GPCR desensitization, the process that blocks continuous G-protein activation by the receptor (Wilden et al., 1986; Lohse et al., 1990; Krupnick and Benovic, 1998; Ferguson, 2001; Moore et al., 2007; Peterson and Luttrell, 2017; Gurevich and Gurevich, 2019a). In the canonical desensitization mechanism, the receptor is first phosphorylated by kinase enzymes, then arrestin binds to the phosphorylated receptor (commonly referred to as arrestin recruitment). This arrestin binding usually sterically blocks G-protein interaction with the receptor, so attenuating G-protein activation by the receptor (Moore et al., 2007; Gurevich and Gurevich, 2019a). Arrestins are also involved in the next event in the desensitization pathway, receptor internalization (Ferguson et al., 1996; Gurevich and Gurevich, 2006; Moore et al., 2007; Shenoy and Lefkowitz, 2011). In this process, arrestins act as scaffolds for proteins involved in endocytosis, e.g., AP2 and calthrin in clathrin-coated pits (Goodman et al., 1996; Laporte et al., 2000). Following internalization, GPCRs in intracellular vesicles are trafficked via two primary pathways, as illustrated in Figure 7 (Gurevich and Gurevich, 2006; Moore et al., 2007; Shenoy and Lefkowitz, 2011). Either the receptor is removed via sorting to degradation compartments, or it is recycled back to the plasma membrane where it can contribute again to G-protein signaling (Oakley et al., 1999, 2000; Zhang et al., 1999; Bremnes et al., 2000; Klein et al., 2001). Arrestins also mediate intracellular signaling by acting as adapter proteins, notably modulation of protein kinase cascades (Luttrell and Gesty-Palmer, 2010; Shenoy and Lefkowitz, 2011; Peterson and Luttrell, 2017; Gurevich and Gurevich, 2019b).

The specific events and pathways mediated by arrestin are controlled by how the arrestin interacts with the GPCR. The interaction is controlled by the pattern of phosphorylation of the GPCR (the phosphorylation barcode) (Orsini et al., 1999; Oakley et al., 2001; Tobin, 2008; Nobles et al., 2011; Pal et al., 2013; Zhou et al., 2017; Sente et al., 2018; Baidya et al., 2020; Dwivedi-Agnihotri et al., 2020; Latorraca et al., 2020) and also by the size and sequence of the intracellular regions of the receptor that form the binding site for arrestin (Oakley et al., 1999; Barak et al., 2001; Thomsen et al., 2016; Chaturvedi et al., 2020). Arrestin interacts with the intracellular face of the GPCR, engaging both the C-terminal tail and the transmembrane core (Shukla et al., 2014; Kang et al., 2015; Nguyen et al., 2019; Yin et al., 2019; Lee et al., 2020; Staus et al., 2020). The binding mechanism has been correlated with the functions that arrestin mediates via the GPCR. For example, tight binding has been associated with internalization and prolonged localization in intracellular compartments (the so-called Type A receptors), whereas looser binding is correlated with shorter intracellular residence time and recycling back to the plasma membrane (Type B receptor) (Figure 7) (Oakley et al., 1999, 2000; Zhang et al., 1999; Bremnes et al., 2000; Klein et al., 2001).

### Evaluating Arrestin Mechanisms From the Arrestin Recruitment Waveform

The time course curve shape, i.e., the waveform, of GPCR signaling can reveal mechanistic insight into the processes of signal transduction and the regulation of signaling events in operation in the cell (Hoare et al., 2018, 2020b). In addition, analyzing the waveform can allow the mechanisms to be quantified kinetically, for example in terms of the rates of the processes. In this study we applied this approach to arrestin recruitment waveforms. We found the waveform shape differed between receptors and this could be accounted for by different regulation pathways, for example degradation vs. recycling. In addition, we identified and quantified the effect of modulators of arrestin-receptor interaction, specifically GPCR kinase (GRK) expression, and the grafting onto the GPCR of the V_2_ vasopressin receptor tail. The new experimental and analysis system developed in this study is designed to be straightforward for investigators to use in studying their receptor systems of interest.

In order to precisely evaluate the waveform, we sought an experimental system, with minimal interference from technical artifacts, that could be run for the time span necessary to properly capture the waveform shape. The direct fluorescent arrestin-3 (β-arrestin 2) biosensor we developed previously is potentially suitable for this application (Hoare et al., 2020a). Notably, it does not involve the use of the unstable substrates that are employed by BRET and luminescent protein complementation sensors that can make it challenging to run these assays for extended periods of time. However, we did need to minimize the baseline drift we had observed in the MOR agonist characterization experiments (Figures 3D,E). To do this, we utilized the BioTek Synergy Mx reader, which enabled control of the stimulation/read frequency. The time interval between stimulation/reads we employed with this instrument was 20 s (compared with 2 s in the MOR agonist characterization experiments on the FDSS/μCell reader). This modification almost eliminated baseline drift, as shown in Supplementary Figure 4. In a second modification, we subtracted the baseline signal from the data before the curve fitting analysis (Supplementary Figure 4). This was done by subtracting baseline data from vehicle control wells run in parallel in each experiment, as described in Materials and Methods. The resulting waveform data was of exceptional quality, with 290 data points spanning a time course of 97 min (Supplementary Figure 4). This enabled precise analysis of the waveform by nonlinear regression (see below).

We evaluated five GPCRs–the V_2_ vasopressin receptor, β_2_ adrenoceptor, μ opioid receptor, NOP nociceptin receptor, and glucagon-like peptide 1 (GLP-1) receptor. These receptors were stimulated using vasopressin, isoproterenol, DAMGO, nociceptin/orphanin FQ(1-13)NH_2_, and exendin-4, respectively. A maximally-stimulating concentration of the agonist was used (10 μM) to enable the response to the fully-occupied receptor to be evaluated. The high agonist concentration also ensures agonist binding to the receptor is not rate limiting; at such high concentrations the receptor is likely fully occupied within seconds by the agonist (Hoare et al., 2020b). In these experiments the arrestin recruitment was optimized by expression of GRK enzymes in the HEK293T cells. In pilot experiments we found arrestin recruitment was maximal when GRK2 was overexpressed in the cells for the β_2_, MOR, NOP and GLP-1 receptors, but that recruitment to the V_2_ receptor was maximal when there was no exogenous expression of GRKs (data not shown). These were the conditions used for the waveform analysis below.

**Figure 8:**
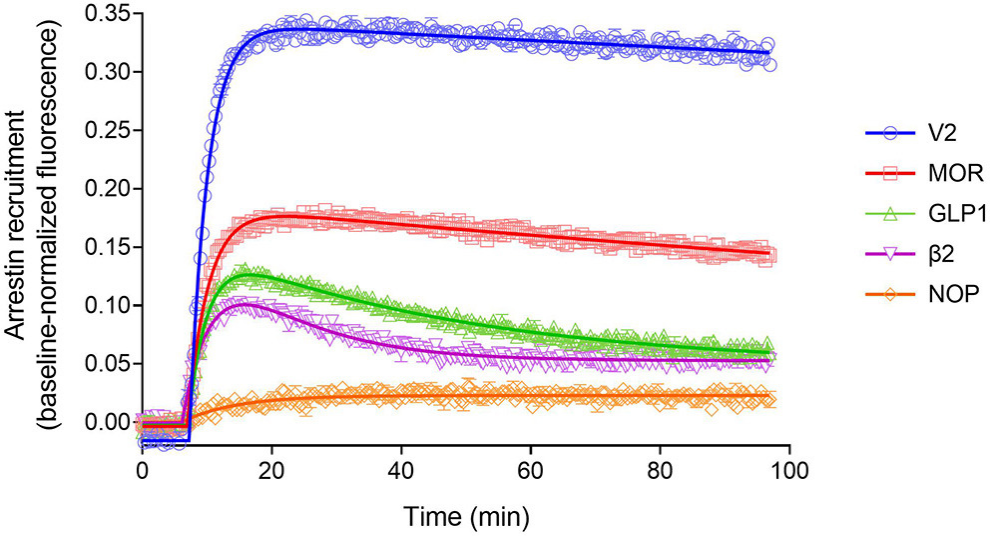
Diversity of arrestin recruitment waveforms for GPCRs. The time course was evaluated for five GPCRs in HEK293T cells, with recruitment optimized by expression of GRK enzymes. Note the different shapes of the waveforms. For the V_2_ vasopressin and μ opioid (MOR) receptors, the waveform rapidly rose to a steady-state then slowly declined. The data were fit best by a model that assumes recruitment followed by slow degradation, representing the degradation pathway in Figure 7. For the GLP-1 and β_2_ adrenergic receptors, the waveform was a rise and fall to steady-state curve, the recruitment rising rapidly, peaking, then falling back down to a steady-state level. The data were fit best by a recruitment followed by recycling model, representing the recycling pathway (Figure 7). For the NOP nociceptin receptor the recruitment rose to a steady-state, the data described best by simple recruitment (over the duration of the experiment). Data points are the mean ± SEM of three technical replicates from a representative experiment, with the curve fitting performed as described in Materials and Methods. GRK2 was expressed in the cells to maximize recruitment for all receptors except the V_2_ receptor. The curves are the fits to the model that fit the data best [recruitment and degradation model (Figure 9B) for V_2_ and MOR receptors; recruitment and recycling model (Figure 9C) for GLP-1 and β_2_ receptors; and recruitment alone model for the NOP receptor (Figure 9A)]. The curve fit parameter values are in Table 3.

The arrestin recruitment waveform for the five GPCRs is shown in Figure 8. Different shapes of the waveform were evident. For the V_2_ vasopressin receptor, reported to form stable complexes with arrestin (a Type A receptor), the arrestin recruitment waveform rapidly rose to a steady-state then slowly declined (Figure 8). By contrast, for the β_2_ adrenoceptor, reported to form transient, recycling complexes with arrestin (a Type B receptor), the waveform was a rise and fall to steady-state curve; the response rose rapidly, then peaked, then declined back to a steady-state level that was above the baseline (Figure 8). This difference suggests the different recruitment mechanisms of Type 1 and Type 2 receptors are manifest in the shape of the waveform.

**Figure 9:**
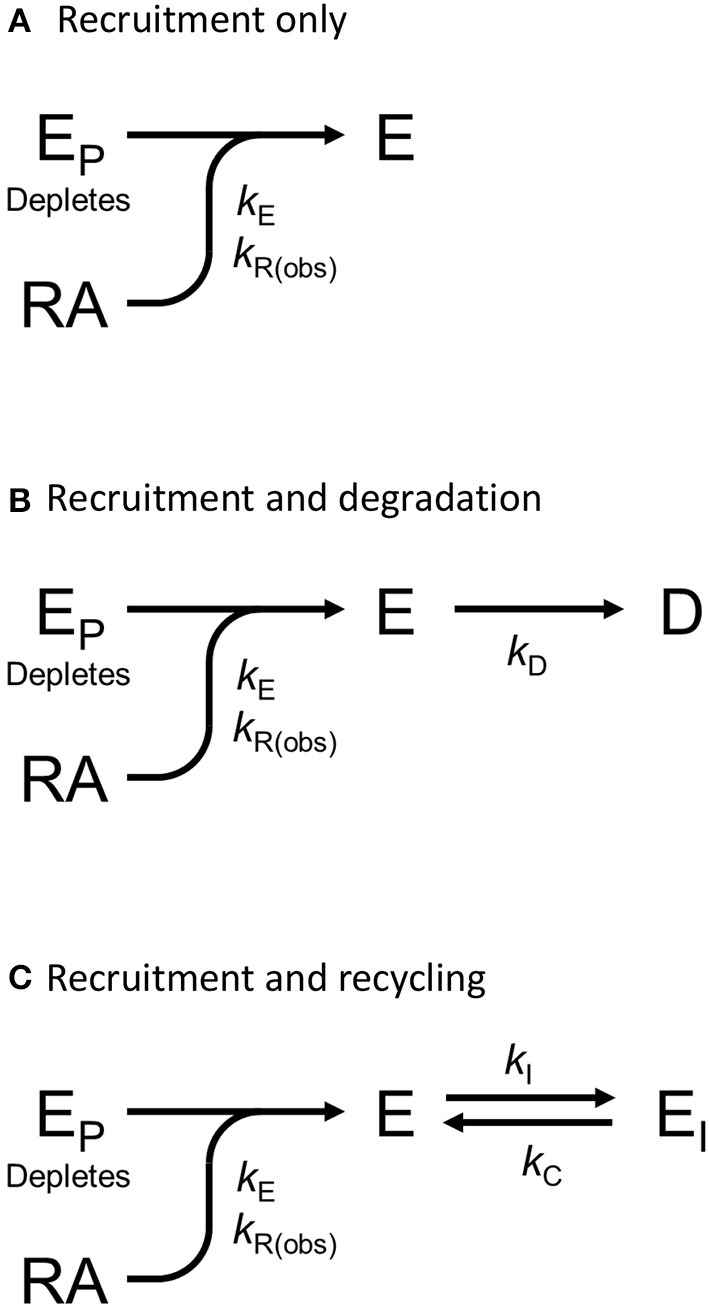
Mechanism schemes used to formulate the arrestin recruitment equations. Simplified pharmacological models of arrestin recruitment and subsequent regulation steps were formulated for analyzing the arrestin waveform data, to evaluate the mechanism in operation and to enable estimation of the macroscopic rates of the processes. These models employ a previously-published conceptual framework (Hoare et al., 2018, 2020b). **(A)** Arrestin recruitment model. E_P_ is free arrestin, E is arrestin bound to the receptor, RA is the receptor bound by the agonist, and *k*_E_ the microscopic rate constant for arrestin binding to the receptor. *k*_R(obs)_ is the observed rate of arrestin recruitment (equal to *k*_E_ multiplied by the agonist-occupied receptor concentration). **(B)** Arrestin recruitment and degradation pathway model. Following recruitment, the complex is degraded, represented by formation of the degraded product D, defined by the rate constant *k*_D_. **(C)** Arrestin recruitment and recycling pathway model. Here the complex breaks down, represented by formation of E_I_, governed by the rate constant *k*_I_. The complex can then recycle, governed by the rate constant *k*_C_.

We tested this more rigorously by deriving equations for the different mechanisms and applying them to the data. The equations used to analyze the data were derived from macroscopic reaction models (Figure 9). This macroscopic approach, frequently used in pharmacological modeling and analysis, allows quantification of the processes in terms of bulk rate constants and steady-state levels of recruitment, using routine curve fitting software such as GraphPad Prism. For example, we were able to quantify the observed arrestin recruitment rate constant (*k*_Robs_) for all the waveforms, and the degradation rate constant *k*_D_ for the degradation waveform. The limitation of the method is that it lacks the high mechanistic resolution of more sophisticated approaches such as systems biology analysis (Bridge et al., 2018); the individual steps in the mechanisms are not quantified and instead are incorporated into macroscopic rates (Hoare et al., 2018, 2020b; Zhu et al., 2019) (For example, receptor phosphorylation and subsequent recruitment are amalgamated into a single rate constant, the observed recruitment rate constant). Stable recruitment is represented in Figure 9A where the arrestin binds the receptor with no further regulation steps. In Figure 9B, the degradation pathway is represented, where the complex forms and then is degraded over time. Figure 9C shows a formulation of the recycling mechanism where there is no degradation and instead the receptor-arrestin complex can reform after breaking down. The waveform data were analyzed with the equations derived from these models (see Appendix for derivation), and the best fit to the experimental data was determined statistically using a partial F-test in Prism (Motulsky, 2021a), as described in Materials and Methods. The V_2_ receptor data were fit best by the degradation model (with no recycling over the duration of the experiment), with rapid recruitment and slow degradation (Figure 8, Table 3). By contrast, the β_2_ adrenoceptor data were fit best by the recycling model (Figure 8, Table 3). These results confirm that the different arrestin mechanisms postulated for these receptors can be manifest as differences in the waveform curve shape.

**Table 3:** Arrestin recruitment waveform quantification results.

**Receptor**	**Recruitment t½** **(*k*_R(obs)_ t½, min)**	**Maximal recruitment** **(% change from baseline)**	**Regulation process**	**Degradation** **t½ (*k*_D_ t½, h)**	**Inactivation t½** **(*k*_I_ t½, min)**	**Recycling t½** **(*k*_C_ t½, min)**
${\text{V}}_{2}^{\text{a}}$	2.5 ± 0.3 (2.0–2.9)	41 ± 3 (36–44)	Degradation	27 ± 8 (14–43)		
MOR	8.3 ± 1.5 (6.4–11)	8.0 ± 0.1 (7.8–8.2)	Degradation	3.2 ± 1.3 (1.9–5.7)		
MOR + GRK2^a^	2.0 ± 0.1^**^ (1.9–2.2)	16 ± 2^*^ (13–19)	Degradation	2.8 ± 0.8^NS^ (1.7–4.3)		
MOR-V_2_ tail	1.8 ± 0.4^**^ (1.3–2.5)	24 ± 2^***^ (22–27)	Degradation	3.7 ± 0.5^NS^ (3.0–4.8)		
NOP	6.6 ± 2.6 (2.2–11)	1.8 ± 0.7 (0.5–3.0)	Recruitment			
NOP + GRK2^a^	3.5 ± 1.1^NS^ (1.5–5.0)	2.1 ± 0.3^NS^ (1.6–2.3)	Recruitment			
NOP-V_2_ tail	1.7 ± 0.3^NS^ (1.2–2.0)	40 ± 2^***^ (35–42)	Degradation	46 ± 33 (3–110)		
β_2_	6.5 ± 0.7 (5.2–7.4)	19 ± 3 (16–26)	Recycling		14 ± 2 (9–17)	16 ± 5 (7–26)
β_2_ + GRK2^a^	3.0 ± 0.2^NS^ (2.6–3.3)	17 ± 1^NS^ (16–20)	Recycling		150 ± 100^NS^ (10–330)	38 ± 7^NS^ (26–51)
β_2_-V_2_ tail	6.9 ± 1.4^NS^ (5.0–9.7)	36 ± 3^*^ (29–39)	Recruitment			
GLP-1	2.7 ± 1.0 (1.8–4.6)	9.3 ± 0.6 (8.1–10)	Recycling		31 ± 8 (19–46)	42 ± 5 (35–52)
GLP-1 + GRK2^a^	2.0 ± 0.3^NS^ (1.6–2.5)	16 ± 2^*^ (14–20)	Recycling		33 ± 5^NS^ (25–44)	110 ± 40^NS^ (60–200)
GLP-1-V_2_ tail	3.5 ± 0.9^NS^ (1.8–4.8)	14 ± 1^NS^ (11–15)	Degradation^b^	3.6 ± 0.1^b^ (3.5–3.6)		

*The waveform data were analyzed using the three macroscopic mechanistic equations/models (recruitment only, degradation pathway, and recycling pathway, Figure 9) and the best fit model determined statistically as described in Materials and Methods. Data values are the mean of values from three independent experiments. Maximal recruitment is the parameter RecruitMax from the curve fitting, which is the plateau recruitment for the recruitment only model, and the extrapolated plateau for the other two models (see Supplementary Figure 5). RecruitMax was expressed as a percentage change from baseline by multiplying the RecruitMax value from the fit by 100. Note the degradation t_1/2_ unit is hours whereas the other t_1/2_ values are in minutes. The effect of GRK2 expression and V_2_ tail grafting, relative to the wild type receptor expression alone, was tested statistically by single-factor ANOVA, followed by Dunnett's multiple comparisons test comparing GRK2 or V_2_ tail with wild type receptor alone. When only two of the three conditions were being compared a two-tailed t test was used*.

NS *, not significant (P > 0.05)*;

* *P < 0.05*;

** *P < 0.01*;

*** *P < 0.001*.

a *Data for conditions used in Figure 8*.

b *In two of three experiments for the GLP-1-V_2_ tail the degradation model fit best whereas in one experiment the recruitment only model fit best. The degradation t_1/2_ data are from the two experiments where the degradation model fit best*.

We next examined the three other receptors. For the MOR receptor with GRK2, the arrestin recruitment waveform resembled that of the V_2_ vasopressin receptor. The waveform rose to a steady-state then slowly declined (Figure 8). The data were fit best by the recruitment followed by degradation model (Table 3). By contrast, for the GLP-1 receptor with GRK2, the waveform was similar to that of the β_2_ adrenoceptor, being a rise and fall to steady-state curve, with the data being fit best by the recycling model (Figure 8, Table 3). For the NOP receptor with GRK2, the extent of recruitment was lower than that of the other receptors. The waveform was a rise to steady-state curve and the data over the duration of the experiment were fit best by a model that assumes recruitment to the receptor without further regulation (Figure 8, Table 3). These findings demonstrate a diversity of arrestin recruitment waveform types, which can be rationalized by differences in the mechanisms of post-recruitment events. These waveform shapes are also apparent from visual inspection of numerous previous studies of arrestin-receptor interaction (Charest et al., 2005; Violin et al., 2006b; Nuber et al., 2016; Gillis et al., 2020a; Dijon et al., 2021), suggesting they are a common feature of arrestin-GPCR interaction.

### Quantifying Arrestin Recruitment by Analyzing the Waveform

The curve fitting also enables quantification of the rates of the processes involved in the mechanisms, enabling these rates to be compared between receptors. In order to compare rates across different mechanisms, the models were formulated with certain common parameters across the different mechanisms, as illustrated in Supplementary Figure 5. The arrestin recruitment rate was quantified in all three models as *k*_Robs_, the observed rate of recruitment. Here this rate is represented as a half-time, which facilitates intuitive interpretation of the data (The initial rate of recruitment could also be determined but was not used here). It was also possible to quantify the steady-state level of recruitment from all three models (Supplementary Figure 5). For the recruitment only model, this was the plateau of the waveform. For the degradation model, this was the extrapolated maximal level of recruitment, and for the recycling model this was the extrapolated maximal level of the initial phase of recruitment, before recycling (illustrated in Supplementary Figure 5). This steady-state level, referred to as RecruitMax, provides an assessment of the affinity of the receptor-arrestin interaction. Finally, it was possible to estimate the rates of the regulation process in the models (*k*_D_, *k*_I_, and *k*_C_). The fitted parameter values are shown in Table 3.

The recruitment half time and maximal recruitment were reliably determined, with the inter-experimental variability of the fitted parameter values (SEM / mean × 100) being <30% in most cases (Table 3). The recruitment half time was similar for all receptors under conditions optimized for GRK expression, the t_1/2_ varying from 2.0 to 3.5 min (Table 3, see rows marked with superscript a). This timing of arrestin recruitment makes sense biologically, being later than the timing of G-protein activation, which proceeds within seconds of agonist binding [see for e.g., Ferrandon et al. (2009)]. The half-time is also within the timeframe of arrestin recruitment detected in numerous previous studies (Charest et al., 2005; Violin et al., 2006b; Nuber et al., 2016; Gillis et al., 2020a; Dijon et al., 2021). The maximal recruitment level varied considerably between the receptors optimized for GRK expression. Recruitment was highest for the V_2_ vasopressin receptor (41% change from baseline) and lowest for the NOP receptor (2.1 %) (Table 3, see rows marked with superscript a). This finding suggests major differences of arrestin-receptor affinity for the different receptors, a phenomenon that is well-established [see for example (Oakley et al., 2000)]. The quantitative analysis provided here enables these differences to be enumerated. For example, the affinity of the arrestin-3 sensor for the V_2_ receptor is 20-fold higher than that for the NOP receptor, in terms of the maximal recruitment value (Table 3).

It was also possible in most cases to reliably quantify the regulation parameters for the later steps of the model mechanisms (Table 3). For the degradation model for the V_2_ vasopressin and MOR receptors, the degradation t_1/2_ was estimated reasonably well with inter-experimental variability (% CV) of ≤ 30%. Degradation for the V_2_ receptor was markedly slow (27 h half-time) whereas that for the MOR was faster (2.8 h). The value for the MOR receptor is within the range of receptor degradation half-times reported for a broad panel of GPCRs in HEK293 cells [0.7–2.8 h (Lee et al., 2021)]. The long half-time for the V_2_ receptor might be a manifestation of the tight arrestin binding impairing degradation. For the recycling model for the β_2_ and GLP-1 receptors the regulation parameters are the inactivation half-time and recycling half-time. Again, these were estimated reasonably well (with the exception of the inactivation half time for the β_2_ receptor where a wide range of values was seen, Table 3). The half time for recycling was 38 min for the β_2_ adrenoceptor (Table 3). This is in range of the reported half-time for dephosphorylation of this receptor in HEK293 cells [~23 min (Tran et al., 2007)]. For the GLP-1 receptor the recycling half-time was slightly longer [110 min, Table 3)].

### Effect of Modifiers of GPCR-Arrestin Interaction on the Arrestin Recruitment Waveform

Experimentally, the effect of modifying GPCR-arrestin interaction on functional outcomes has been explored by manipulating the recruitment interaction. This has been done by replacing receptor sequences with higher-affinity determinants of arrestin interaction, notably substituting the C-terminal tail of the GPCR with that of a receptor that stably interacts with arrestin, e.g., the V_2_ vasopressin receptor (Oakley et al., 1999, 2000; Zhang et al., 1999; Pal et al., 2013; Thomsen et al., 2016). In addition, the strength of arrestin-receptor interaction and functional consequences can be manipulated by modifying the expression of GRK subtypes (Kim et al., 2005; Ren et al., 2005; Violin et al., 2006b), which presumably modifies receptor phosphorylation. Here the effect of these manipulations on the arrestin recruitment waveform was evaluated, to determine whether changes in the interaction could be manifest as changes of the shape of the waveform and changes of the rates of the processes.

**Figure 10:**
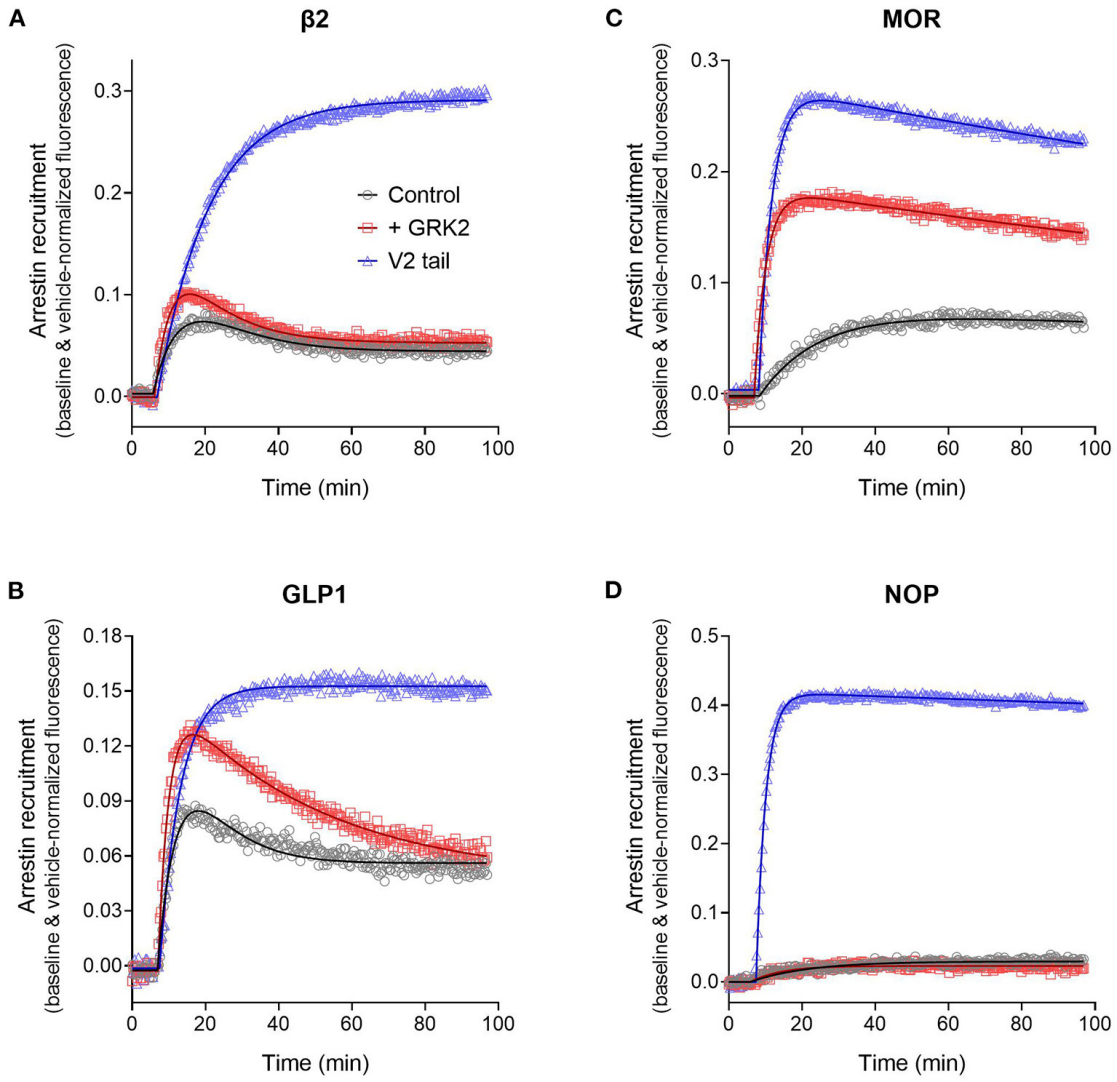
Effect of arrestin recruitment and function modifiers on the arrestin recruitment waveform. The strength and mechanism of arrestin recruitment can be affected by the sequence of the receptor, particularly the C-terminal tail, and by expression of receptor kinase enzymes, assumed to modulate receptor phosphorylation. This was explored by grafting the V_2_ vasopressin receptor C-tail onto the receptors, a determinant of high-affinity, stable arrestin interaction, and by expression of GRK2, as described in Materials and Methods. **(A)** β_2_ adrenoceptor. **(B)** GLP-1 receptor. **(C)** μ opioid receptor. **(D)** NOP nociceptin receptor. Data points are the mean ± SEM of three technical replicates from a representative experiment. The curves are fits to the arrestin recruitment equations and the specific arrestin model/equation curve type is listed in Table 3.

We first evaluated the effect of substituting the C-terminal tail of the GPCRs with that of the V_2_ vasopressin receptor, as described (Oakley et al., 2000). The last 29 C-terminal amino acids of the V_2_ receptor were substituted into the β_2_, GLP-1, MOR and NOP receptors (see Materials section in Materials and Methods). In this experiment, the waveform for the wild-type and V_2_ tail receptors was measured in the absence of exogenous GRK enzyme expression in the cells. This was done because GRK2 expression decreased the signal for the V_2_ receptor and for V_2_ tail receptors (data now shown). The results clearly show the V_2_ tail determined the waveform shape (Figure 10). For the β_2_ and GLP-1 receptors, the V_2_ tail changed the shape from a rise and fall to steady-state curve (wild type control) to a rise to steady-state curve (V_2_ tail, Figures 10A,B, Table 3) (For the GLP-1 receptor, there was a slow decline after reaching the plateau, Figure 10B). This shape is similar to that of the V_2_ receptor (Figure 8). When fit to the mechanistic equations, the V_2_ tail changed the waveform from the recycling model to the recruitment only model (β_2_ receptor) or the degradation pathway model (GLP-1 receptor). The V_2_ tail also substantially increased maximal recruitment for the β_2_ receptor (from 19 to 36%, Table 3), suggesting an increased affinity of the arrestin-receptor interaction. There was also a numerical increase of maximal recruitment for the GLP-1 receptor, but the difference was not statistically significant (Table 3).

For MOR and NOP receptors, the V_2_ tail did not change the shape of the waveform, as expected since for all three wild-type receptors the waveform shape was similar (V_2_ in Figure 8, MOR and NOP in Figures 10C,D). However, the V_2_ tail did substantially increase maximal recruitment for MOR and NOP receptors (Figures 10C,D, Table 3), suggesting an increased affinity of the interaction. The rate of recruitment (recruitment half-time) was not significantly affected by the V_2_ tail for β_2_, GLP-1, and NOP receptors (Table 3), suggesting the increased maximal recruitment for these receptors was a result of a slower rate of dissociation of the complex, a mechanism invoked previously (Oakley et al., 2000). This analysis of the waveforms provides supporting evidence that the arrestin–receptor C-terminal tail interaction is a determinant of the strength and regulatory mechanism of GPCR-arrestin interaction, as proposed previously (Oakley et al., 1999, 2000; Zhang et al., 1999; Pal et al., 2013; Thomsen et al., 2016). The analysis also provides quantitative insight into the magnitude of the effects. For example, the effect of the V_2_ C-tail on maximal recruitment, presumed to reflect the affinity for arrestin, varied from 1.5-fold for the GLP-1 receptor to 22-fold for the NOP receptor.

We next evaluated the effect of phosphorylation on the arrestin recruitment waveform by differential expression of GRKs. Recruitment was compared with and without transduction of GRK2, which was found in pilot experiments to be the GRK subtype that most affected arrestin recruitment for the receptors under test (data not shown). The effect of GRK2 was different from that of the V_2_ tail in that GRK2 expression did not change the shape of the waveform but instead changed either the rate of recruitment and/or the maximal recruitment for most of the receptors (Figure 10, Table 3). This was most evident for the MOR receptor (Figure 10C) where GRK2 expression significantly increased both the rate (manifest as a reduced half-time) and the maximal recruitment (Table 3). For the GLP-1 receptor, GRK2 expression significantly increased the maximal recruitment. For β_2_ and NOP receptors the recruitment half-time was reduced by GRK2 expression but the difference was not statistically significant (Table 3).

This increased rate and extent of recruitment upon GRK expression and/or receptor phosphorylation has been observed previously (Wilden et al., 1986; Gurevich et al., 1993; Sohlemann et al., 1995; Violin et al., 2006b) and can be readily explained by the mechanism of recruitment (Figure 7). In the canonical mechanism, receptor phosphorylation precedes arrestin binding and phosphorylation is the rate limiting step. The effect on the rate of recruitment can then be explained simply by mass action, by the presumably greater amount of receptor kinase activity resulting from GRK2 expression resulting in an increased rate of receptor phosphorylation and subsequent arrestin recruitment. Receptor phosphorylation also increases the affinity of receptor-arrestin interaction and this was manifest in the case of MOR and GLP-1 receptors as an increase of the maximal recruitment (Table 3).

### Summary

In this study we demonstrated that measuring and analyzing the waveform for arrestin recruitment could indicate mechanisms of arrestin function and enable the kinetics to be rigorously quantified. This required two advances. First, an assay was required that could be run for sufficient time for the whole waveform to be captured (90 min) and this was achieved using a very bright direct fluorescent biosensor which did not require the use of unstable light-generating substrates. Careful control of the plate reader settings, particularly the stimulation/read frequency, minimized photobleaching, and the resulting waveform was of exceptional quality. The second advance was the development of equations for analyzing the data. These new equations enable macroscopic evaluation of the different arrestin recruitment and functional mechanisms and can be applied to the data in familiar curve fitting software (e.g., GraphPad Prism). The waveform analysis provided confirmatory evidence for the hypothesis of varying strengths and mechanisms of arrestin interaction with the different GPCRs, and how these properties are affected by the C-terminal tail and by receptor kinase expression. The waveform analysis enables these differences to be quantified in terms of intuitive parameters, such as the recruitment half-time, maximal recruitment, and degradation or recycling half-times. This provides an advance over previous, largely qualitative studies that relied on visual inspection of the data in most cases [with some exceptions, e.g., Oakley et al. (2000)]. The assay and analysis described here will facilitate future quantitative research on the dynamics of arrestin recruitment and function.

## Summary and Concluding Remarks

In this study we have developed systems to quantify the signaling kinetics of GPCRs involved in important drug and receptor responses in the nervous system, including the opioid and CB_1_ receptor, and arrestin recruitment by numerous nervous system GPCRs. These systems can be applied in future studies to measure signaling kinetics. Notably, the present studies were conducted using transfected cells (HEK293 and AtT20) and the approach developed here has not been formally applied to the receptors in their native environment, e.g., in neurons. Experimental conditions necessary to apply the analysis are often not employed in biosensor experiments performed on neuronal and other native cells. Frequently in experiments using these cell types the agonist is applied for a short time and then washed out before the waveform has been properly defined. The analysis method presented in this study requires the continuous application of the agonist and for the signal to be recorded long enough for the curve shape to be defined rigorously. Other technical aspects to be considered are the temperature; in the biosensor experiments in this study room temperature (21–22°C) was used for technical convenience but the dynamics are likely to be temperature dependent. Finally, the amount of sensor needs to be titrated to ensure sufficient signal but without signal saturation, as described previously (Hoare and Hughes, 2021).

New fluorescent biosensors are being discovered at a remarkable rate. They provide us with exciting, real time views of signaling that until now were studied with end point assays. They also reveal entirely new cellular phenomena such as the recent discovery of phase transitions in the cytosol (Hyman and Simons, 2012; Watanabe et al., 2017; Zhang et al., 2021a). These new views come with new challenges. In the field of Ca^2+^ signaling, for example, the combination of transgenic animals and the biosensors drove the innovation behind new kinds of microscopy and circuit analysis (Ahrens et al., 2013; Sofroniew et al., 2016). Now that it is possible to measure and follow real time GPCR signaling through multiple pathways, the next challenges are how to collect the relevant kinetic data over the correct time and space scales, and most importantly, how to extract meaning from these new, very rich data sets.

## Materials and Methods

### Materials

The cDNA for the β_2_ adrenergic, GLP-1, MOR, NOP, and V_2_ vasopressin receptors, and the cDNA for GRK2, was obtained from the cDNA Resource Center (Bloomsburg University, Bloomsburg, PA). In some experiments receptors modified to include the C-terminal tail of the V_2_ vasopressin receptor were used. This involved substituting the last C-terminal residues with the last 29 C-terminal residues of the V_2_ receptor (Oakley et al., 2000) (last 72 amino acids of the β_2_ adrenoceptor, and the last 29 amino acids of the MOR, NOP and GLP-1 receptors). DAMGO, met-enkephalin, endomorphin-2, morphine, hydromorphone, oxymorphone, fentanyl, buprenorphine, vasopressin, N/OFQ and exendin-4 were all obtained from Cayman Chemical (Ann Arbor, MI). Isoproterenol was from Millipore Sigma.

### Biosensor Assays for Quantifying μ-Opioid Receptor cAMP Signaling and Arrestin Recruitment

Genetically-encoded biosensors in the BacMam expression system were used to measure cAMP inhibition and arrestin recruitment via the μ opioid receptor. The sensors have been described previously (Green Downward cADDis for cAMP (Tewson et al., 2016, 2018), and the arrestin-3 (β-arrestin 2) sensor (Hoare et al., 2020a). The experiments were conducted in HEK293T cells transduced with the receptor and each sensor individually (i.e., one batch of cells with receptor and the cAMP sensor and a separate batch with receptor and arrestin sensor). HEK 293T cells were cultured in Eagle's minimum essential media (EMEM) supplemented with 10% fetal bovine serum and penicillin-streptomycin at 37 °C in 5% CO_2_. One day before the transduction, HEK293T cells were seeded at a density of 8,000 cells/well in a volume of 50 μL on a Greiner CELLCOAT 384-well-black, clear-bottomed plate (Greiner Cat # 781946, Millipore Sigma).

The next day, cells were transduced with either the Green Downward cADDis or β-arrestin sensor BacMam stocks. To prepare the transduction mixture, the BacMam containing the cADDis or arrestin-3 sensor and the indicated receptors, 2 mM sodium butyrate, and EMEM were combined in a final volume of 50 μL. For each cADDis experiment, 1.8 × 10^8^ viral genes of cADDis virus and 5.9 × 10^7^ viral genes of MOR virus were added to each well. For each arrestin experiment, 6.7 × 10^7^ viral genes of arrestin virus, 2.0 × 10^7^ viral genes of GRK2 virus and 7.0 x 10^7^ viral genes of MOR virus were added to each well. The transduction mixture was then added to the 384-well-plate (50 μL/well) and incubated for ~24 h at 37°C in 5 % CO_2_.

The assays were performed at room temperature (21–22°C). Prior to fluorescence plate reader experiments, the media in each well was replaced with 35 μL of Dulbecco's phosphate buffered saline (DPBS) supplemented with Ca^2+^ (0.9 mM) and Mg^2+^ (0.5 mM). This was done 60 min prior to the cAMP experiment and 30 min prior for the arresin experiment. For the cAMP assay, cells were pre-incubated with 20 μM forskolin for ~50 min before application of MOR agonists, a time interval sufficient for a steady-state plateau level of cAMP to be reached [see Figure 5 of Hoare and Hughes (2021)]. Test compounds were prepared and serially diluted in the appropriate vehicle (water for met-enkepahalin and endomorphin 2, and DMSO for DAMGO, morphine, hydromorphone, oxymorphone, fentanyl and buprenorphine). The serial dilution factor was 4.64 and the final DMSO assay concentration was 0.1%. Fluorescence plate reader experiments were performed on the Hamamatsu FDSS/μCell Functional Drug Screening System (Hamamatsu Photonics). The green fluorescence detection was recorded using 470 nm excitation and 540 nm fluorescence emission. Baseline fluorescence prior to application of the agonist was recorded for 4 min, then MOR agonists were applied to all wells simultaneously in a volume of 10 μL using the on-board automated dispensing system. All wells were scanned simultaneously, with the exposure time for the cADDis sensor being 0.36 to 0.4 s, while the exposure time for the arrestin sensor was 0.64 to 0.71 s. Each scan was performed every 2 s. Every fifth data point was used in the data analysis, corresponding to a time point interval in the analysis of 10 s. This represented a reasonable trade-off between precision and data file size. Duplicate technical replicates were used.

### Biosensor Assay for Measuring Arrestin Recruitment Waveform for Various Receptors

The fluorescent arrestin-3 (β-arrestin 2) sensor (Hoare et al., 2020a) was used to investigate the shape of the waveform for a variety of receptors. The experiments were conducted in HEK293T cells using the BacMam expression system. Cells were transduced with the receptor of interest, the arrestin sensor, and in some cases the kinase GRK2. HEK 293T cells were cultured in EMEM supplemented with 10% fetal bovine serum and penicillin-streptomycin at 37 °C in 5 % CO_2_ For BacMam transduction, cells were resuspended in media at a density of 52,000 cells per 100 μL. 100 μL of this suspension was combined with BacMam containing the arrestin-3 sensor, receptor, 2 mM sodium butyrate, and EMEM in a final volume of 150 μL per well. In some cases, GRK2 was also added. The receptors tested were the V_2_ vasopressin receptor, β_2_ adrenoceptor, μ opioid receptor, NOP nociceptin receptor, and GLP-1 receptor. Each well-received 3.0 × 10^8^ viral genes of the arrestin sensor, 4.5 × 10^8^ viral genes of the receptor, and 1.2 × 10^8^ viral genes of GRK2, if included. The cell/transduction mixture was then seeded into Greiner CELLCOAT 96-well-black, clear-bottomed plate (Greiner cat # 655946, Millipore Sigma) and incubated for 24 h at 37 °C in 5% CO_2_.

The assays were performed at room temperature (21–22°C). Prior to fluorescence plate reader experiments, the media in each well was replaced with 150 μL of DPBS supplemented with Ca^2+^ (0.9 mM) and Mg^2+^ (0.5 mM). This was done 30 min before the experiment. Test compounds were prepared in the appropriate vehicle - isoproterenol in 10 mM HCl for the β_2_ adrenoceptor, vasopressin in water for the V_2_ receptor, DAMGO in DMSO for the MOR, N/OFQ in water for the NOP receptor, and exendin-4 in DPBS for the GLP-1 receptor. Vehicle was diluted 1,000-fold into the assay. A maximally-effective concentration of the ligands was employed (10 μM in all cases). Three technical replicates were employed for the agonist condition and two replicates used for the vehicle condition. The appropriate vehicle was run in each assay and was used to correct the fluorescent signal (see below).

Fluorescence was measured in the BioTek Synergy Mx reader (Agilent). Green fluorescence detection was recorded every 20 s using 488/20 nm excitation and 525/20 nm fluorescence emission. Following recording of baseline fluorescence for 6 min, agonist was added manually with a multichannel pipette in a volume of 50 μL and the fluorescence recorded for an addition 90 min.

### Membrane Potential Assay for the CB_1_ Receptor

Time course data for the reduction of membrane potential stimulated by CB_1_ receptor ligands is from Sachdev et al. (2019). This response was measured in AtT20 cells stably transfected with the human CB_1_ receptor. The change in membrane potential is mediated by endogenous GIRK channels in the cells, likely activated by the G-protein βγ dimer released from G-protein following activation by the agonist-bound receptor [reviewed in Sachdev et al. (2019)]. The level of receptor expression was reduced to maximize the window for detecting differences of agonist efficacy for activation of this signaling pathway. This was done by treating the cells with the irreversible antagonist AM6544 as described (Sachdev et al., 2019). Changes in membrane potential were measured using the fluorometric imaging plate reader (FLIPR) membrane potential (blue) assay kit (Molecular Devices) at 37 °C as previously described (Knapman et al., 2013; Sachdev et al., 2019). A maximally-effective concentration of the agonist was used (see Table 2 for concentration values).

### Data Handling

The raw fluorescence measurement recorded by the plate readers was fluorescence intensity, in units of relative light units. These raw data were normalized to the baseline response prior to application of the agonist, as described (Hoare and Hughes, 2021). This approach is commonly used for fluorescent readouts of signaling activity and provides an ideal intra-well-control, minimizing well-to-well variability of the signal readout resulting from slight differences in the amount of sensor or the number of cells. All experiments were designed with a baseline run in period where the baseline fluorescence in the well was measured prior to activation of signaling by application of the GPCR agonist (see for example Figure 1B). The mean fluorescence intensity value of this baseline run-in period was used as the baseline value and the fluorescence intensity value at each time point was divided by this value. This yielded the baseline-normalized fluorescence value. This value was used for the membrane potential measurements for the CB_1_ receptor. A second step was performed for the downward cADDis sensor for cAMP and for the arrestin sensor. Binding of cAMP to the cADDis sensor and binding of receptor to the arrestin sensor results in a decrease in fluorescence intensity of the biosensor. For these data, the baseline-normalized value was inverted by subtracting it from 2, as described (Hoare and Hughes, 2021). This results in the directionality of the normalized fluorescence value being the same as that of the signaling analyte or event being detected.

For the arrestin sensor recruitment measured using the BioTek Synergy Mx reader the baseline fluorescence measured using a vehicle control was subtracted from the agonist-stimulated fluorescence, as illustrated in Supplementary Figure 5. This was done using the “Remove baseline and column math” functionality of GraphPad Prism (Motulsky, 2021b). In this procedure, the vehicle time course was assumed to be linear and the vehicle data were fit to a straight line function. For each time point the vehicle value was then calculated from the straight line fit parameters and this value was then subtracted from the agonist value, to give the vehicle and baseline-normalized fluorescence value (Y axis value in Figures 8, 10).

### Curve Fitting and Calculation of Initial Rate

#### General

Time course data were analyzed using user-defined custom equations in GraphPad Prism. These have been made freely available for other investigators to use at the following location: https://drive.google.com/drive/folders/1F5Qlyi30a3VNu9ZzCTKuTCDEmH6B4rdX?usp=sharing. A user guide is provided in the file, “Custom time course equations background info” at this location. We have also created a training workshop, available here: https://youtu.be/_Pb7Sq6lZIY. The equations can be easily loaded automatically from template files, avoiding the need to manually enter the equation, and these template files also contain the default initial value calculations and constraints. This process of equation loading is described in the file, “Guide for loading equations into Prism from a file.” The template files can be found at the location given above.

#### μ Opioid cAMP Inhibition and Arrestin Recruitment Time Course Data Analysis

The MOR cAMP inhibition data were analyzed with the equation “Baseline then fall to steady state with drift” which is:


$$\begin{array}{l} \text{Y}=\text{IF }\left(\begin{array}{c} \text{X}<\text{X}0,\text{Baseline}+\text{Drift}\times \text{X},\\ \text{YS}+\text{Drift}\times \left(\text{X}-\text{X}0\right)-\text{SteadyState}\times \left(1-{\text{e}}^{-\text{K}\times \left(\text{X}-\text{X}0\right)}\right) \end{array}\right) \end{array}$$

where:


$$\begin{array}{l} \text{YS}=\text{Baseline}+\text{Drift }\times \text{X}0 \end{array}$$

Y is the baseline-normalized fluorescence signal, X is time, X0 is the signal start time after the application of agonist, Baseline is the response level at the beginning of the fluorescence recording in the reader, Drift is the gradient of the baseline drift (in units of Y units per unit time), SteadyState is the final effect level at infinite time produced by ligand below the baseline, and K is the observed rate constant, which defines the timeframe over which the cAMP inhibition occurs and is related to the half-time of the response (t_1/2_ = 0.693 / K). The initial rate of the response was calculated automatically as part of the fitting procedure in Prism, using the formula: Initial rate = SteadyState × K.

The MOR arrestin recruitment data were analyzed with the equation “Baseline then rise to steady state with drift” which is:


$$\begin{array}{l} Y=\text{IF }\left(\begin{array}{c} \text{X}<\text{X}0,\text{Baseline}+\text{Drift}\times \text{X},\\ \text{YS}+\text{Drift}\times \left(\text{X}-\text{X}0\right)+\text{SteadyState}\times \left(1-{\text{e}}^{-\text{K}\times \left(\text{X}-\text{X}0\right)}\right) \end{array}\right) \end{array}$$

where the parameters are defined as described above for the cAMP inhibition analysis. The initial rate of the response was calculated automatically as part of the fitting procedure in Prism, using the calculation Initial rate = SteadyState × K.

#### CB_1_ Membrane Potential Time Course Data Analysis

The CB_1_ membrane potential time course data for all ligands except THC were analyzed with the equation “Baseline then fall to steady state time course” which is,


$$\begin{array}{l} Y=\text{IF }\left(\begin{array}{c} \text{X}<\text{X}0,\text{Baseline},\\ \text{Baseline}-\text{SteadyState}\times \left(1-{\text{e}}^{-\text{K}\times \left(\text{X}-\text{X}0\right)}\right) \end{array}\right)\; \end{array}$$

where the parameters are defined as described above for the cAMP inhibition analysis. In the CB_1_ membrane potential experiment, there was a significant injection artifact manifest as a small, immediate drop in the normalized fluorescence signal in the vehicle and THC-treated cells (Figure 5). For the vehicle, this was analyzed using the following step-function equation to quantify the magnitude of the drop:


$$\begin{array}{l} Y=\text{IF }\left(\begin{array}{c} \text{X}<\text{X}0,\text{Baseline},\\ \text{Baseline}-\text{Step}+\text{Gradient }\times \left(\text{X}-\text{X}0\right)\; \end{array}\right)\; \end{array}$$

where Step is the magnitude of the immediate normalized fluorescence signal change on application of the vehicle, and Gradient is the change of the vehicle response over time after X0. The value of Step was highly reproducible between experiments (ranging from 4.2 to 5.0 RFU, *n* = 6, see Supplementary Material “CB_1_ hyperpolarization time course fit results”).

For THC the response was relatively small and slow compared with the other ligands and as a result the injection artifact was evident in the time curve shape (Figure 5). For this ligand an equation was used that combined the injection artifact and the pharmacological effect of the agonist on membrane potential:


$$\begin{array}{l} \text{Y}=\text{IF }\left(\begin{array}{c} \text{X}<\text{X}0,\text{Baseline},\\ \text{Baseline}-\text{Step}-\text{SteadyState}\times \left(1-{\text{e}}^{-\text{K}\times \left(\text{X}-\text{X}0\right)}\right) \end{array}\right)\; \end{array}$$

The initial rate of membrane potential reduction was calculated as follows, for all ligands except THC. First, the SteadyState value from the curve fit was corrected for the injection artifact. This was done by subtracting the mean Step value of the vehicle (4.4 %) from the fitted SteadyState value. The corrected SteadyState value was then combined with the K value from the curve fit to determine the initial rate, using the formula: Initial rate = SteadyState_(Corrected)_ × K. For THC, the SteadyState value fitted from the curve fit equation used was already corrected for the injection artifact so the initial rate was calculated using the standard equation, Initial rate = SteadyState × K.

#### Arrestin Recruitment Waveform Analysis

The waveform of arrestin recruitment was analyzed for numerous GPCRs under a variety of conditions. The time course data were fit to the arrestin recruitment time course equations described in the Appendix. In order to assess which model/equation fit the data best, a statistical procedure was used. The data were fit to the equations and the equation that fit the data best was determined using a partial F-test, using the “Compare” function in the “Non-linear regression” module of GraphPad Prism (Motulsky, 2021a).

The arrestin recruitment equations were entered as user-defined equations in GraphPad Prism. A Prism template file containing the equations is available from the authors on request. The “Arrestin recruitment” (Figure 9A) equation is,


$$\begin{array}{l} \text{Y}=\text{IF }\left(\begin{array}{c} \text{X}<\text{X}0,\text{Baseline},\\ \text{Baseline}+\text{RecruitMax}\times \left(1-{\text{e}}^{-\text{KRobs}\times \left(\text{X}-\text{X}0\right)}\right) \end{array}\right)\; \end{array}$$

The “Arrestin recruitment and degradation” (Figure 9B) equation is,


$$\begin{array}{l} \text{Y}=\text{IF }\left(\begin{array}{c} \text{X}<\text{X0},\text{ Baseline},\\ \text{Baseline}+\frac{\text{InitialRate}}{\text{KRobs}-\text{KD}}\times \left({\text{e}}^{-\text{KD}\times \left(\text{X}-\text{X0}\right)}-{\text{e}}^{-\text{KRobs}\times \left(\text{X}-\text{X0}\right)}\right) \end{array}\right) \end{array}$$

The “Arrestin recruitment and recycling” (Figure 9C) equation is,


$$\begin{array}{l} \text{Y}=\text{IF }(\text{X}<\text{X}0,\text{Baseline},\text{ Baseline}\\ +\;\frac{\text{Initial rate }\times \text{ KC}}{\text{KRobs }\left(\text{KC}+\text{KI}\right)}[1-\frac{\text{KC}+\text{KI}}{\text{KC}+\text{KI}-\text{KRobs}}e^{-\text{KRobs}\times \left(\text{X}-\text{X}0\right)}\\ +\frac{\text{KRobs}}{\text{KC}+\text{KI}-\text{KRobs}}e^{-\left(KC+KI\right)\times \left(\text{X}-\text{X}0\right)}]\\ +\frac{\text{Initial rate}}{\text{KC}+\text{KI}-\text{KRobs}}\left({\text{e}}^{-\text{KRobs}\times \left(\text{X}-\text{X}0\right)}-{\text{e}}^{-\left(\text{KC}+\text{KI}\right)\times \left(\text{X}-\text{X}0\right)}\right)) \end{array}$$

Y is the baseline- and vehicle-normalized fluorescence signal, X is time, X0 is the signal start time after the application of agonist, Baseline is the response level before application of agonist, RecruitMax is the change of fluorescence stimulated by the agonist at infinite time, *k*_Robs_ is the observed rate of arrestin recruitment, Initial rate is the initial rate of recruitment, *k*_D_ is the degradation rate constant of the degradation model, and *k*_I_ and *k*_C_ are the inactivation and recycling rate constants, respectively, of the recycling model. For reporting purposes the rate constant were converted to half times. This was done by dividing ln 2 (0.693) by the rate constant value.

From the degradation and recycling models the extrapolated steady-state level of arrestin recruitment of the first, rising phase of the time course (RecruitMax) was calculated (Supplementary Figure 5). This was done using the following equation:


$$\begin{array}{l} \text{RecruitMax}=\frac{\text{InitialRate}}{\text{KRobs}} \end{array}$$

This equation is the limit of the degradation and recyling equations when *k*_D_, *k*_C_ and *k*_I_ are set to zero and time is set to infinity (illustrated in Supplementary Figure 5). RecruitMax from the fit, where the Y axis is baseline and vehicle-normalized fluorescence, was converted to % change from baseline by multiplying the fit value by 100.

### Statistical Analysis

Differences of fitted parameter values between different conditions were tested statistically by single factor ANOVA, followed by the Dunnett multiple comparison test comparing the test conditions to the relevant control. When two conditions were being compared a two-tailed *t*-test was used. Statistical analysis was performed using GraphPad Prism.

## Data Availability Statement

The raw data supporting the conclusions of this article will be made available by the authors, without undue reservation.

## Author Contributions

SRJH analyzed data and wrote the manuscript. PHT and SS performed experiments and analyzed data. MC, TEH, and AMQ conceived the study and wrote the manuscript. All authors contributed to the article and approved the submitted version.

## Funding

Opioid and arrestin research reported in this publication was supported by National Institute of General Medical Sciences, National Institutes of Health under Award Number R44GM125390, and National Institute on Drug Abuse, National Institutes of Health R44NS082222. Cannabinoid research was supported by National Health and Medical Research Council Project Grant 1107088, National Institutes of Health Grant P01DA009158, and the European Union's Seventh Framework Program (FP7/2007-103, Grant Agreement HEALTH-F2-2011-278850 and R21DA045882).

## Conflict of Interest

SRJH was employed by the company Pharmechanics LLC. PHT, TEH, and AMQ were employed by Montana Molecular. The remaining authors declare that the research was conducted in the absence of any commercial or financial relationships that could be construed as a potential conflict of interest.

## Publisher's Note

All claims expressed in this article are solely those of the authors and do not necessarily represent those of their affiliated organizations, or those of the publisher, the editors and the reviewers. Any product that may be evaluated in this article, or claim that may be made by its manufacturer, is not guaranteed or endorsed by the publisher.

## Abbreviations

DPBS: Dulbecco's phosphate buffered saline
EMEM: Eagle's minimum essential media
GPCR: G-protein-coupled receptor
GRK2: G-protein-coupled receptor kinase 2
G IR_max_: maximal initial rate
N/OFQ: nociceptin/orphanin FQ(1-13)NH_2_
SCRA: synthetic cannabinoid receptor agonist.
