# Supplementary material for: Quantifying the Kinetics of Signaling and Arrestin Recruitment by Nervous System G-Protein Coupled Receptors
Source: Front Cell Neurosci. 2022 Jan 17;15:814547. doi: 10.3389/fncel.2021.814547 (PMC8801586; doi:10.3389/fncel.2021.814547)
Supplement: Supplementary file 1 [file Data_Sheet_1.zip › Supplementary Material Documents/Appendix.docx]

# Appendix

## Curve shape equations

Time course data for GPCR signaling is usually described by one of four shapes, as described in the section “Curve fitting for time course signaling data” (Hoare et al., 2020b). These are the straight line, the rise to steady-state curve, the rise-and-fall to baseline curve, and the rise-and-fall to steady-state curve. Each shape is defined by a specific equation, as follows:

**Straight line time course.** See Figure 2A. This is the familiar linear regression equation, where t is time:

$$Signal=t \mathrm{Slope}$$

Eqn. 1

**Rise to steady-state time course.** See Figure 2B. This is the association exponential equation:

$$Signal=SteadyState \left( 1-e^{-K.t} \right)$$

Eqn. 2

where SteadyState is the level of signal at infinite time and K is the observed rate constant in units of t^-1^. K defines the timeframe over which the cAMP inhibition occurs and is related to the half-time of the response (t_1/2_  = 0.693 / K).

**Rise and fall to baseline time course.** See Figure 2C:

$$Signal=\frac{C}{K_{1}-K_{2}} \left( e^{-K_{2}.t}-e^{-K_{1}.t} \right)$$

Eqn. 3

where C is a fitting constant in units of signal units.t^-1^ (which is the initial rate of signaling, see below) and K_1_ and K_2_ are observed rate constants related to the rise phase and fall phase in units of t^-1^. Again, K_1_ and K_2_ can be represented as half times by dividing 0.693 by the rate constant value.

**Rise and fall to steady-state time course.** See Figure 2D:

$$Signal=SteadyState \left( 1-De^{-K_{1}.t}+\left( D-1 \right)e^{-K_{2}.t} \right)$$

Eqn. 4

where SteadyState is the final signal level at infinite time, D is a unitless fitting constant, and K_1_ and K_2_ are observed rate constants related to the rise phase and fall phase in units of t^-1^.

## Formulas for calculating the initial rate of signal generation.

Traditionally, determining the initial rate, the rate of the first, linear portion of the time course, has involved manually assessing which data points lie on this portion of the curve. We developed an automated procedure that avoids this process and utilizes all the time points of the time course curve. The parameters from the curve fit are entered into a formula that calculates the initial rate. These formulas were derived by taking the limit of the time course equations as time approaches zero (Hoare et al., 2020b). The formulas are as follows:

**Straight line time course:**

$$Initial rate=Slope$$

Eqn. 5

**Rise to steady-state time course:**

$$Initial rate=SteadyState K$$

Eqn. 6

**Rise and fall to baseline time course:**

$$Initial rate=C$$

Eqn. 7

**Rise and fall to steady-state time course:**

$$Initial rate=SteadyState \left( DK_{1}-\left( D-1 \right)K_{2} \right)$$

Eqn. 8

## GraphPad Prism equations for analyzing experimental time course data

Under normal experimental conditions, there are additional components to the analysis to consider that we incorporated into the suite of equations we developed for use in GraphPad Prism. First, there is the baseline signal in the absence of agonist. Second, in many experiments this baseline signal is measured for a period of time before application of the agonist. Third, some signals go down instead of up, for example inhibition of cAMP generation by Gi-coupled receptors (see Figure 3A,B). Finally, sometimes there is an upward or downward drift of the baseline signal, which can result from slight photobleaching of fluorescent biosensors and from luminescent substrate decay for luminescent and BRET-based biosensors. We incorporated these features into the suite of equations for use in GraphPad Prism. In the Supplementary File, “Time course equation list” these equations are shown together with graphs showing the curve shapes and the meaning of the parameters. Prism templates containing the equations can be downloaded from this location: <https://drive.google.com/drive/u/1/folders/1F5Qlyi30a3VNu9ZzCTKuTCDEmH6B4rdX>.

## Arrestin recruitment time course analysis equations

The mechanisms of arrestin recruitment and subsequent steps in the receptor regulation pathway are well established and are shown in Figure 7. From this general scheme we developed simplified pharmacological models and equations that could be used to fit the arrestin recruitment time course data (Figure 9). Three models were developed that could describe the three curve shapes observed experimentally. In order of increasing complexity, these are the arrestin recruitment model, the recruitment and degradation pathway model, and the recruitment and recycling pathway model. The model framework and equations are based on previously formulized models (Hoare et al., 2018;Hoare et al., 2020b).

### Arrestin recruitment model

In this model, arrestin is recruited to the receptor in a stable interaction without any further regulation steps (like degradation or recycling). This model is represented schematically in Figure 9A, where E_P_ is free arrestin, E is arrestin bound to the receptor, RA is the receptor bound by the agonist, and *k*_E_ the microscopic rate constant for arrestin binding to the receptor. The model assumes the level of free arrestin is depleted by formation of the receptor-arrestin complex, in agreement with experimental data since complex formation results in a substantial change of baseline sensor fluorescence (Hoare et al., 2020a). The model also assumes the complex does not deplete the level of free agonist-occupied receptor, in order to accommodate the observation that expression of the arrestin sensor does not result in significant changes in G-protein-mediated signaling (unpublished observations). The differential equation defining E, the signal being measured, is,

$$\frac{dE}{dt}=E_{P}\left[ \mathrm{RA} \right]k_{E}$$

EP can be expressed in terms of the total amount of E using a conservation of mass equation:

$$E_{\mathrm{TOT}}=E_{P}+E$$

Substituting and rearranging gives,

$$\frac{dE}{dt}=E_{\mathrm{TOT}}\left[ \mathrm{RA} \right]k_{E}-E\left[ \mathrm{RA} \right]k_{E}$$

This equation is now integrated to give,

$$E_{t}=E_{\mathrm{TOT}}\left( 1-e^{-\left[ \mathrm{RA} \right]k_{E}t} \right)$$

This can be reduced to a simpler empirical form for fitting to the data, as follows:

$$E_{t}=\mathrm{RecruitMax}\times\left( 1-e^{-k_{R(obs)}t} \right)$$

where *k*_R(obs)_ is the observed rate constant for arrestin recruitment (equal to $\left[ \mathrm{RA} \right]\times k_{E}$), and RecruitMax is the arrestin recruitment at steady-state. The formula defining the initial rate of arrestin recruitment can be obtained from these equations, as the limit as time approaches zero, which is,

$$Initial rate=E_{\mathrm{TOT}}\left[ \mathrm{RA} \right]k_{E}=RecruitMax\times k_{R(obs)}$$

### Arrestin recruitment and degradation

Subsequent to formation, the arrestin-receptor complex follows two pathways. In the first, the complex is trafficked towards degradation compartments and in the second it is recycled. The degradation pathway can be represented by a degradation step, defined by the rate constant *k*_D_, as shown in Figure 9B. A time course equation defining a mechanism of this type has been derived previously (see Model 4 in (Hoare et al., 2018). Applying the derivation to this system gives the following time course equation:

$$E_{t}=\frac{E_{\mathrm{TOT}}\left[ \mathrm{RA} \right]k_{E}}{\left[ \mathrm{RA} \right]k_{E}-k_{D}}\left( e^{-k_{D}t}-e^{-\left[ \mathrm{RA} \right]k_{E}t} \right)$$

This equation can be rewritten in a more convenient empirical form. As shown above, $E_{\mathrm{TOT}}\left[ \mathrm{RA} \right]k_{E}$ is the initial rate of arrestin recruitment, and $\left[ \mathrm{RA} \right]k_{E}$ is *k*_R(obs)_, the observed rate constant for arrestin recruitment. Inserting these terms gives

$$E_{t}=\frac{Initial rate}{k_{R(obs)}-k_{D}}\left( e^{-k_{D}t}-e^{-k_{R(obs)}t} \right)$$

### Arrestin recruitment and recycling

The recycling pathway can be represented schematically by a separation of the complex followed by reformation, as shown in Figure 9C. Here the complex E breaks down, represented by formation of E_I_, governed by the rate constant *k*_I_. The complex can then recycle, governed by the rate constant *k*_C_. A time course equation defining a mechanism of this type has been derived previously (see the “Precursor depletion & response degradation to steady-state” model in (Hoare et al., 2020b)). Applying the derivation to this system gives the following time course equation:

$$E_{t}=\frac{E_{\mathrm{TOT}}\left[ \mathrm{RA} \right]k_{E}k_{C}}{\left[ \mathrm{RA} \right]k_{E}\left( k_{C}+k_{I} \right)}\left[ 1-\frac{k_{C}+k_{I}}{k_{C}+k_{I}-\left[ \mathrm{RA} \right]k_{E}}e^{-\left[ \mathrm{RA} \right]k_{E}t}+\frac{\left[ \mathrm{RA} \right]k_{E}}{k_{C}+k_{I}-\left[ \mathrm{RA} \right]k_{E}}e^{-\left( k_{C}+k_{I} \right)t} \right]+\frac{E_{\mathrm{TOT}}\left[ \mathrm{RA} \right]k_{E}}{k_{C}+k_{I}-\left[ \mathrm{RA} \right]k_{E}}\left( e^{-\left[ \mathrm{RA} \right]k_{E}t}-e^{-\left( k_{C}+k_{I} \right)t} \right)$$

This equation can be rewritten in a more convenient empirical form. As shown above, $E_{\mathrm{TOT}}\left[ \mathrm{RA} \right]k_{E}$ is the initial rate of arrestin recruitment, and $\left[ \mathrm{RA} \right]k_{E}$ is *k*_R(obs)_, the observed rate constant for arrestin recruitment. Inserting these terms gives:

$$E_{t}=\frac{Initial rate \times k_{C}}{k_{R(obs)}\left( k_{C}+k_{I} \right)}\left[ 1-\frac{k_{C}+k_{I}}{k_{C}+k_{I}-k_{R(obs)}}e^{-k_{R(obs)}t}+\frac{k_{R(obs)}}{k_{C}+k_{I}-k_{R(obs)}}e^{-\left( k_{C}+k_{I} \right)t} \right]+\frac{Initial rate}{k_{C}+k_{I}-k_{R(obs)}}\left( e^{-k_{R(obs)}t}-e^{-\left( k_{C}+k_{I} \right)t} \right)$$

## References

Hoare, S.R.J., Pierre, N., Moya, A.G., and Larson, B. (2018). Kinetic operational models of agonism for G-protein-coupled receptors. *J Theor Biol* 446**,** 168-204.

Hoare, S.R.J., Tewson, P.H., Quinn, A.M., and Hughes, T.E. (2020a). A kinetic method for measuring agonist efficacy and ligand bias using high resolution biosensors and a kinetic data analysis framework. *Sci Rep* 10**,** 1766.

Hoare, S.R.J., Tewson, P.H., Quinn, A.M., Hughes, T.E., and Bridge, L.J. (2020b). Analyzing kinetic signaling data for G‑protein‑coupled receptors. *Scientific Reports* 10**,** 12263.
