# Supplementary material for: Quantifying the Kinetics of Signaling and Arrestin Recruitment by Nervous System G-Protein Coupled Receptors
Source: Front Cell Neurosci. 2022 Jan 17;15:814547. doi: 10.3389/fncel.2021.814547 (PMC8801586; doi:10.3389/fncel.2021.814547)
Supplement: Supplementary file 1 [file Data_Sheet_1.zip › Supplementary Material Documents/Supplementary Figure 2.pdf]

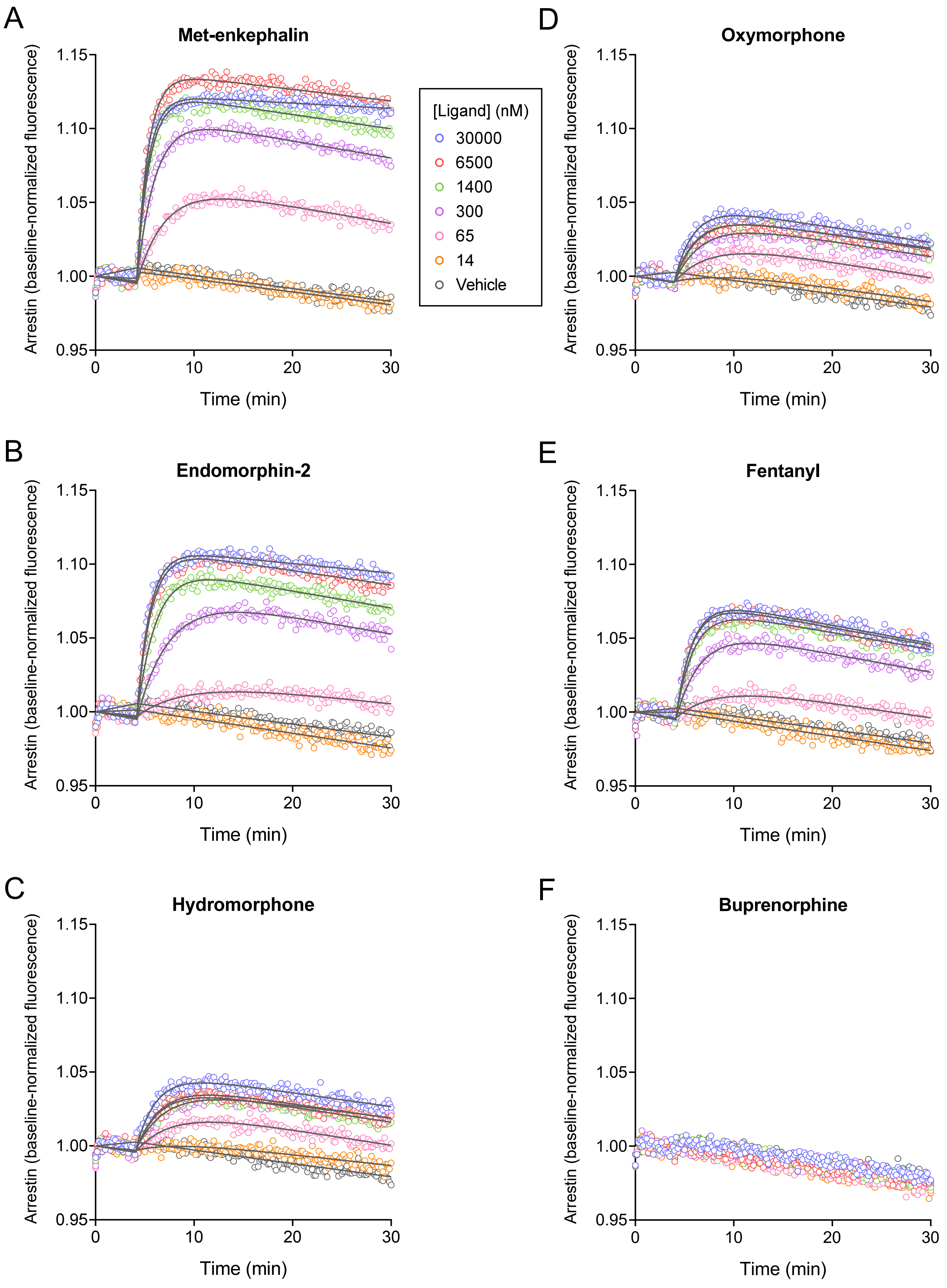

Supplementary Figure 2. Time course of arrestin recruitment to the μ-opioid receptor stimulated by numerous agonist ligands. (Note DAMGO and morphine were also tested - see Figure 3.) Data are from a representative experiment and data points are the mean of two technical replicates. The grey lines are fits to the "Baseline then rise to steady state with drift" equation.
