# Supplementary material for: Quantifying the Kinetics of Signaling and Arrestin Recruitment by Nervous System G-Protein Coupled Receptors
Source: Front Cell Neurosci. 2022 Jan 17;15:814547. doi: 10.3389/fncel.2021.814547 (PMC8801586; doi:10.3389/fncel.2021.814547)

Supplementary Figure 3. Time course data for CB1-mediated membrane potential reduction by various CB1 agonists. Membrane potential was measured using a fluorescent dye in AtT20 cells and data normalized to the baseline signal before application of agonist (10 μM) or vehicle. Data were analyzed by curve fitting to fall to steady-state equations as described in Materials and Methods.Data points are the mean of two technical replicates and data are from representative experiments.

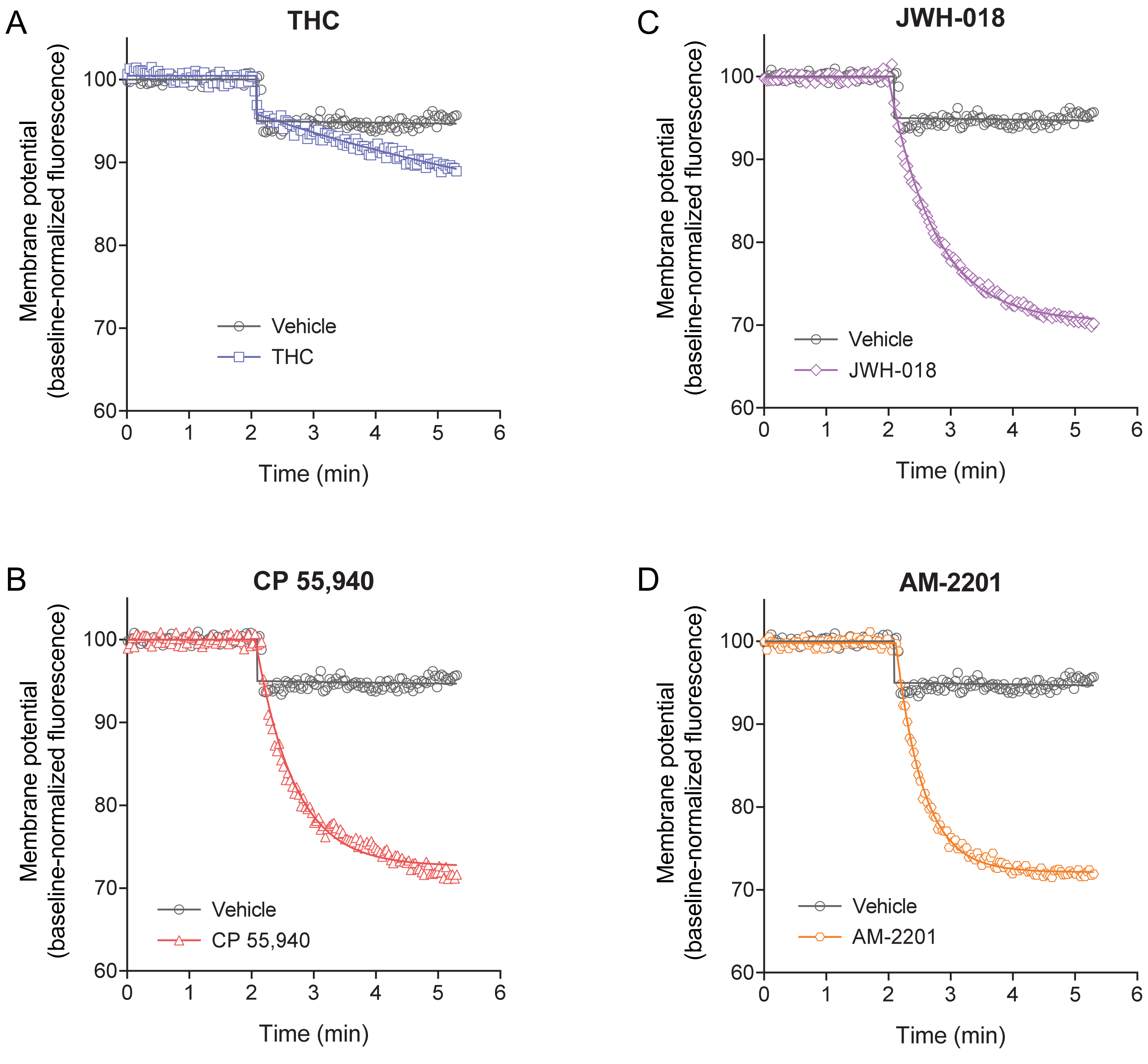

Supplementary Figure 3. continued

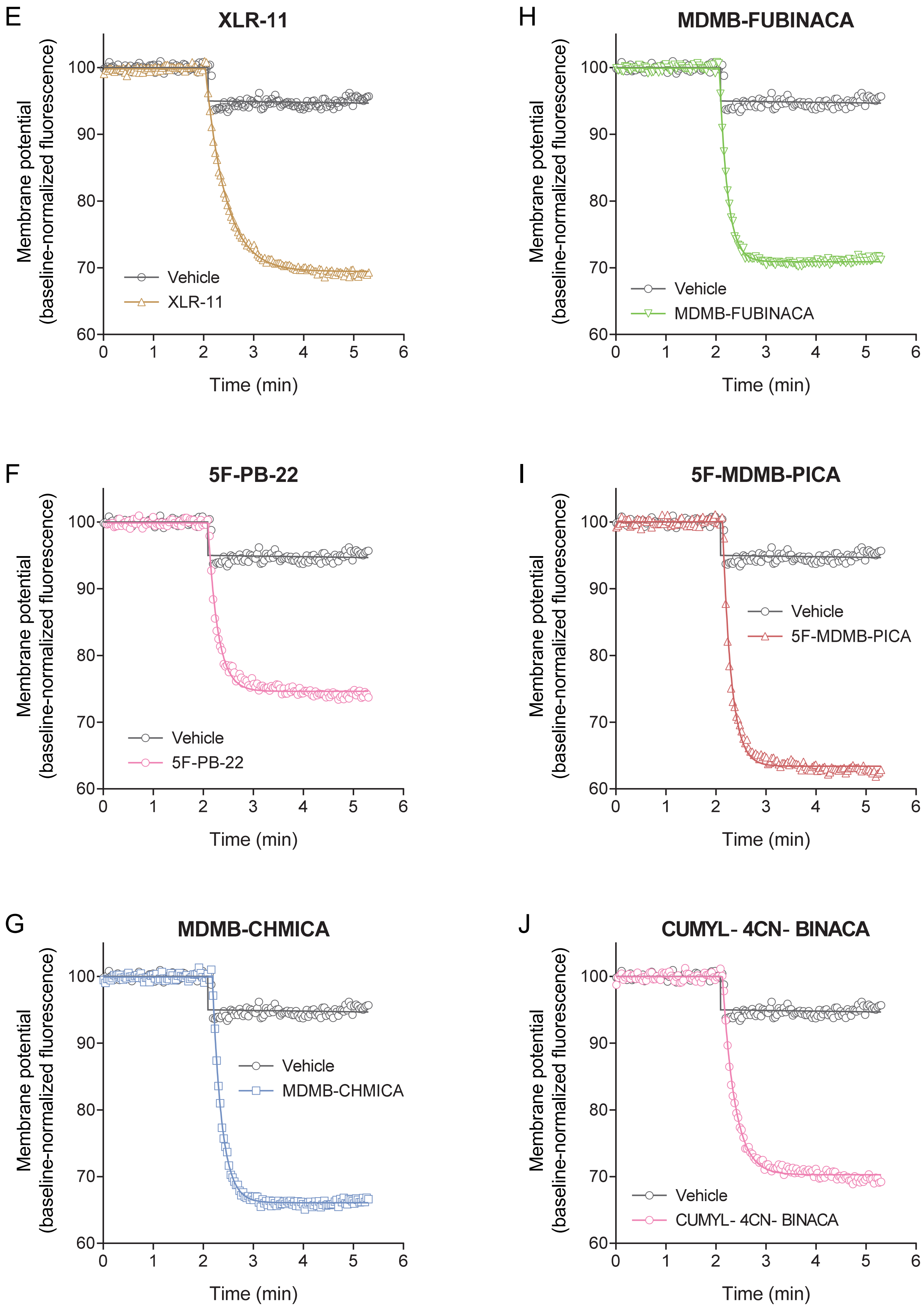

Supplement: Supplementary file 1 [file Data_Sheet_1.zip › Supplementary Material Documents/Supplementary Figure 3.pdf]
