# Supplementary material for: Quantifying the Kinetics of Signaling and Arrestin Recruitment by Nervous System G-Protein Coupled Receptors
Source: Front Cell Neurosci. 2022 Jan 17;15:814547. doi: 10.3389/fncel.2021.814547 (PMC8801586; doi:10.3389/fncel.2021.814547)
Supplement: Supplementary file 1 [file Data_Sheet_1.zip › Supplementary Material Documents/Supplementary Figure 4.pdf]

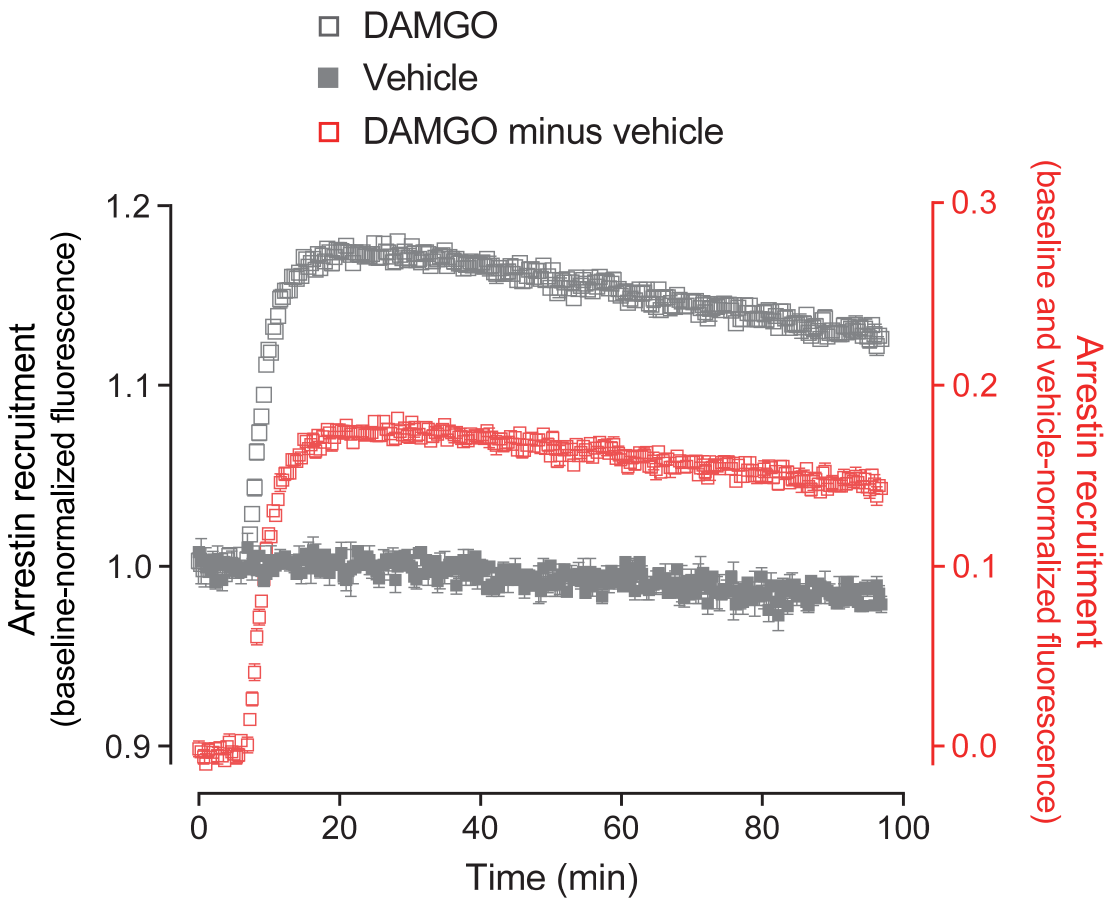

Supplemental Figure 4. Vehicle correction method for measuring arrestin recruitment on the BioTek Synergy Mx reader. In each experiment, a vehicle control was included. The time course of fluorescence intensity was measured for the agonist condition and the vehicle condition. These data were normalized to the baseline fluorescence measured in the first six mintes before application of agonist or vehicle (left-hand Y axis, grey symbols) as described in Materials and Methods. Next, the vehicle and baseline-normalized fluorescence was subtracted from that of the agonist condition, to provide the baseline and vehicle-normalized fluorescence value (right-hand Y axis, red symbols). This was done using the "Remove baseline and column math" functionality of GraphPad Prism (https://www.graphpad.com/guides/prism/latest/user-guide/using_removebaseline.htm?q=remove+baseline). In this procedure, the vehicle time course was assumed to be linear and the vehicle data were fit to a straight line function. For each time point the vehicle value was then calculated from the straight line fit parameters and this value was then subtracted from the agonist value, to give the vehicle and baseline-normalized fluorescence value (Y axis value in Figures 7 and 8).
