# Supplementary material for: Quantifying the Kinetics of Signaling and Arrestin Recruitment by Nervous System G-Protein Coupled Receptors
Source: Front Cell Neurosci. 2022 Jan 17;15:814547. doi: 10.3389/fncel.2021.814547 (PMC8801586; doi:10.3389/fncel.2021.814547)
Supplement: Supplementary file 1 [file Data_Sheet_1.zip › Supplementary Material Documents/Supplementary Figure 5.pdf]

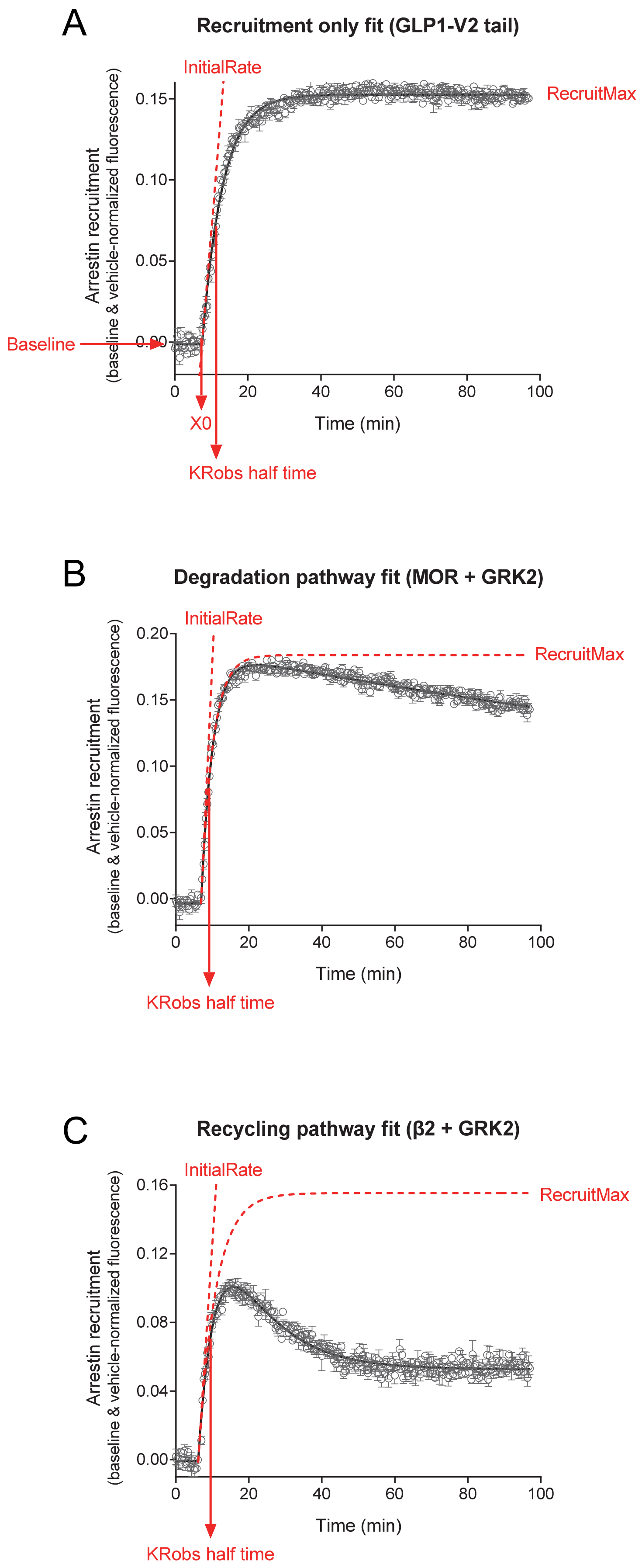

Supplementary Figure 5. Curve fit parameters for the three arrestin recruitment model equations. A) Arrestin recruitment only model, illustrating X0 (the signal start time), Baseline (the signal level before application of agonist), InitialRate (the gradient of the intial rate of recruitment), KRobs half-time (recruitment half time, equal to ln(2) / KRobs), and RecruitMax (the recruitment level at infinite time). B) Degradation pathway model. C) Recycling pathway model. Note RecruitMax is the maximal level of the recruitment phase for the degradation and recycling pathway models. See Materials and Methods for equations.
