# Supplementary material for: Quantifying the Kinetics of Signaling and Arrestin Recruitment by Nervous System G-Protein Coupled Receptors
Source: Front Cell Neurosci. 2022 Jan 17;15:814547. doi: 10.3389/fncel.2021.814547 (PMC8801586; doi:10.3389/fncel.2021.814547)
Supplement: Supplementary file 1 [file Data_Sheet_1.zip › Supplementary Material Documents/Supplementary Table 1.docx]

**Supplementary Table 1. μ opioid receptor signaling initial rate concentration-response parameters.** The initial rate was plotted versus the agonist concentration, for inhibition of cAMP accumulation and stimulation of arrestin recruitment (Figure 4). The data were fit to a sigmoid curve equation (see Materials and Methods) to measure the parameters indicated in the table. Note IR_max_ is the span of the curve (Top minus Bottom) and L_50_ is the half-maximally-effective agonist concentration. Values are the mean ± SEM of values from two independent experiments. ND – not detected.

| Agonist | cAMP inhibition | | | | Arrestin recruitment | | | |
| --- | --- | --- | --- | --- | --- | --- | --- | --- |
|  | IR_max_  (% DAMGO) | -log L_50_  (Log M) | L_50_  (nM) | Hill slope | IR_max_  (% DAMGO) | -log L_50_  (Log M) | L_50_  (nM) | Hill slope |
| DAMGO | 100 | 8.42 ± 0.19 | 3.8 | 0.54 ± 0.09 | 100 | 6.39 ± 0.05 | 400 | 1.04 ± 0.13 |
| Met-enkephalin | 90 ± 10 | 8.99 ± 0.06 | 1.0 | 0.70 ± 0.25 | 97 ± 2 | 6.58 ± 0.06 | 270 | 1.16 ± 0.08 |
| Endomorphin-2 | 96 ± 1 | 8.22 ± 0.04 | 6.1 | 0.77 ± 0.02 | 71 ± 2 | 6.16 ± 0.08 | 690 | 1.03 ± 0.11 |
| Morphine | 103 ± 7 | 8.33 ± 0.12 | 4.7 | 0.65 ± 0.04 | 33 ± 4 | 6.28 ± 0.03 | 530 | 0.78 ± 0.06 |
| Hydromorphone | 94 ± 5 | 8.74 ± 0.29 | 1.8 | 0.97 ± 0.19 | 22 ± 3 | 6.89 ± 0.01 | 130 | 1.06 ± 0.14 |
| Oxymorphone | 106 ± 16 | 8.91 ± 0.20 | 1.2 | 0.63 ± 0.11 | 26 ± 6 | 6.74 ± 0.20 | 180 | 0.79 ± 0.11 |
| Fentanyl | 107 ± 16 | 8.68 ± 0.08 | 2.1 | 0.75 ± 0.23 | 46 ± 4 | 6.58 ± 0.02 | 180 | 1.15 ± 0.08 |
| Buprenorphine | 78 ± 4 | 7.19 ± 0.02 | 65 | 1.69 ± 0.31 | ND | ND | ND | ND |
