# Supplementary material for: Quantifying the Kinetics of Signaling and Arrestin Recruitment by Nervous System G-Protein Coupled Receptors
Source: Front Cell Neurosci. 2022 Jan 17;15:814547. doi: 10.3389/fncel.2021.814547 (PMC8801586; doi:10.3389/fncel.2021.814547)
Supplement: Supplementary file 1 [file Data_Sheet_1.zip › Supplementary Material Documents/Time course equation list.docx]

# Time course equation list

1. Y is the signal being measured
2. X is time
3. These are user-defined custom equations. Instructions on how to install a user-defined equation from a template file are provided [here](https://drive.google.com/drive/folders/1F5Qlyi30a3VNu9ZzCTKuTCDEmH6B4rdX?usp=sharing).
4. The baseline signal is included in the equations. Baseline is signal in the absence of agonist.
5. Multiple equation formats to handle multiple baseline scenarios are presented.

- No baseline run-in period. Signal starts at X = 0.
- Baseline run-in period. Signal starts after X = 0.
- Baseline run-in period with baseline drift. Handles the scenario where baseline drifts over time.

# Rise-and-fall equations

## Rise-and-fall to baseline time course

Y=(C/(K1-K2))*(exp(-K2*X)-exp(-K1*X))+Baseline

## Baseline then rise-and-fall to baseline time course

Y=IF(X<X0, Baseline, Baseline+(C/(K1-K2))*(exp(-K2*(X-X0))-exp(-K1*(X-X0))))

## Baseline then rise-and- fall to baseline with drift

YS=Baseline+Drift*X0

Y=IF(X<X0, Baseline+Drift*X, YS + Drift*(X-X0) + (C/(K1-K2))*(exp(-K2*(X-X0))-exp(-K1*(X-X0))))

## Rise-and-fall to steady state time course

Y=SteadyState*(1-D*exp(-K1*x)+(D-1)*exp(-K2*x))+Baseline

## Baseline then rise-and-fall to steady state time course

Y=IF(X<X0, Baseline, Baseline+SteadyState*(1-D*exp(-K1*(X-X0))+(D-1)*exp(-K2*(X-X0))))

## Baseline then rise-and-fall to steady state with drift

YS=Baseline+Drift*X0

Y=IF(X<X0, Baseline+Drift*X, YS + Drift*(X-X0) + SteadyState*(1-D*exp(-K1*(X-X0))+(D-1)*exp(-K2*(X-X0))))

# Fall-and-rise equations

## Fall-and-rise to baseline time course

Y=(-C/(K1-K2))*(exp(-K2*X)-exp(-K1*X))+Baseline

## Baseline then fall-and-rise to baseline time course

Y=IF(X<X0, Baseline, Baseline+(-C/(K1-K2))*(exp(-K2*(X-X0))-exp(-K1*(X-X0))))

## Baseline then fall-and-rise to baseline with drift

YS=Baseline+Drift*X0

Y=IF(X<X0, Baseline+Drift*X, YS + Drift*(X-X0) + (-C/(K1-K2))*(exp(-K2*(X-X0))-exp(-K1*(X-X0))))

## Fall-and-rise to steady state time course

Y=(-SteadyState)*(1-D*exp(-K1*x)+(D-1)*exp(-K2*x))+Baseline

## Baseline then fall-and-rise to steady state time course

Y=IF(X<X0, Baseline, Baseline+(-SteadyState)*(1-D*exp(-K1*(X-X0))+(D-1)*exp(-K2*(X-X0))))

## Baseline then fall-and-rise to steady state with drift

YS=Baseline+Drift*X0

Y=IF(X<X0, Baseline+Drift*X, YS + Drift*(X-X0) + (-SteadyState)*(1-D*exp(-K1*(X-X0))+(D-1)*exp(-K2*(X-X0))))

# Rise to steady-state equations

## Rise to steady state time course

Y=SteadyState*(1-exp(-K*X))+Baseline

## Baseline then rise to steady state time course

Y=if(X<X0, Baseline, SteadyState*(1-exp(-K*(X-X0)))+Baseline)

## Baseline then rise to steady state with drift

YS=Baseline+Drift*X0

Y=IF(X<X0, Baseline+Drift*X, YS + Drift*(X-X0) + SteadyState*(1 - exp(-K*(X-X0))))

# Fall to steady state equations

## Fall to steady state time course

Y=Baseline-SteadyState*(1-exp(-K*X))

## Baseline then fall to steady state time course

Y=if(X<X0, Baseline, Baseline–SteadyState*(1-exp(-K*(X-X0))))

## Baseline then fall to steady state with drift

YS=Baseline+Drift*X0

Y=IF(X<X0, Baseline + Drift*X, YS + Drift*(X-X0) - SteadyState*(1 - exp(-K*(X-X0))))

# Straight line equations

## Straight line time course

Y=Baseline+Slope*X

## Baseline then straight line time course

Y=IF(X<X0, Baseline, Baseline+Slope*(X-X0))

## Baseline then straight line with drift

YS=Baseline+Drift*X0

Y=IF(X<X0, Baseline+Drift*X, YS + Drift*(X-X0) + Slope*(X-X0))
